# Ethnobotanical study of medicinal plants in Ganta Afeshum District, Eastern Zone of Tigray, Northern Ethiopia

**DOI:** 10.1186/s13002-018-0266-z

**Published:** 2018-11-03

**Authors:** Leul Kidane, Gebrecherkos Gebremedhin, Tadesse Beyene

**Affiliations:** 1 Department of Biology, College of Natural and Computational Sciences, Mekelle University, P.O.Box 231, Mekelle, Ethiopia; 2 Agazi Preparatory School, P.O.Box 014, Adigrat, Eastern Zone of Tigray, Northern Ethiopia Ethiopia

**Keywords:** Diversity, Ethnomedicine, Extinction, Ganta Afeshum, Medicinal plant, Sustainable use, Tigray

## Abstract

**Background:**

Starting from the ancient time, the people of Ethiopia use medicinal plants as traditional medicine to heal different human and livestock ailments. This ethnobotanical study of medicinal plants was carried out in Ganta Afeshum District, Eastern Zone of Tigray, Northern Ethiopia, to identify medicinal plant species used by the local community to treat various human and livestock ailments.

**Methods:**

A total of 78 informants (54 men and 24 women) were selected to collect ethnobotanical information from four study sites. Among the 78 informants, 20 key informants were selected purposefully; the other 58 informants were selected randomly by lottery method. Ethnobotanical data were collected using semi-structured interviews, field observations, guided field walks, and group discussions and were analyzed by preference ranking, paired comparison, direct matrix ranking, informant consensus factor, fidelity level (FL), use-value, independent samples *t* test, and Pearson correlation coefficients.

**Results:**

A total of 173 medicinal plants were collected and identified that were distributed across 77 families and 156 genera. The family Fabaceae stood first by contributing 17 (9.8%) species followed by Lamiaceae and Solanaceae with 9 (5.2%) species each. *Rhamnus prinoides* was reported for the treatment of many of the described diseases. One hundred sixteen (67.1%) medicinal plant species were collected from natural vegetation, 34 (19.7) were from home gardens, 13 (7.5%) from farmland, and 10 (5.8%) were from natural vegetation and home gardens. The most widely used life form was herbs (69 species, 39.9%) followed by shrubs (58 species, 33.5%). The most commonly used part of the medicinal plants was the leaves followed by roots. The plants were prepared by grinding, powdering, squeezing, roasting, and burning and were administered through oral, dermal, nasal, anal, ocular, and vaginal, and on the surface of the teeth. The most commonly used applications were by drinking, smearing, eating, fumigation, and chewing. There was no difference between men and women informants, showing that the two sexes had similar knowledge in the use of traditional medicinal plants. Educational level and medicinal plant knowledge of informants were negatively correlated; whereas age and medicinal plant knowledge of informants were positively correlated.

**Conclusions:**

Ganta Afeshum District is relatively rich in diversity of medicinal plant resources accompanied with a rich indigenous knowledge within the local communities to harvest and effectively use to prevent different human and livestock ailments. However, nowadays, deforestation, agricultural expansion, overgrazing, drought, and overexploitation are threatening these properties. Therefore, people of the study area should apply complementary conservation approaches (in situ and ex situ) for sustainable use of these resources and to prevent species extinction.

**Electronic supplementary material:**

The online version of this article (10.1186/s13002-018-0266-z) contains supplementary material, which is available to authorized users.

## Background

Humans began to employ plants for the intention of health a long time ago, maybe at the first moment when they suffered from diseases [1]. Since the antique time plants have been an essential supply for deterrent and healing for humans and livestock [2]. The population living in Sub-Saharan Africa continues to suffer from infectious as well as noninfectious and deficiency diseases [3]. Because of these and other problems, a large number of people of Africa die daily of preventable and curable diseases due to the lack of simple primary health care [4].

The ailment saddle is provoked by the limitation of the medical personnel and medical provisions such as remedial devices and access to fundamental medicine. The ratio of medical doctors to patients in Africa is not fair; in Ethiopia, for example, there is one doctor to 33,000 patients and in Malawi one doctor to 50,000 patients [5]. Because of this, human beings use different plant species known in ancient traditional medicine instead. Traditional medicine has been applied by humans for the healing of different diseases since a long time before the beginning of conventional medicine and up to this time serve the health care needs of the majority of the people of Africa [3, 5–7].

Thus, traditional medicine remains popular for both historical and cultural reasons. It is estimated that 80% of the African people depend on traditional medicine to meet up their care needs [8].

Like other parts of sub-Saharan countries, 70% of human and 90% of livestock population of Ethiopia rely on traditional medicine for primary health care [9]. In addition to the lack of availability of modern medicine, there are also culturally linked traditions. The communities have trust in the medicinal values of traditional medicine which can also be obtained at a relatively low cost as compared to the modern ones [10].

Ethiopia is exceptionally rich in history, culture, and biological diversity. It is the origin of the early of hominine species of which Lucy was a member. Around 80 languages are spoken by various ethnic groups. The country is also recognized for its diverse habitats, vegetation, and faith which results in a high diversity of traditional medicinal knowledge and practices of the people in using medicinal plants [11]. However, this rich cultural heritage is threatened, especially in the form of deforestation, fuelwood collection, illegal logging, overgrazing by stock animals, and agricultural expansion [11–13]. Such problems include the Tigray Region where the study was conducted.

Although the literature on ethnobotany in Ethiopia is increasing, there is still a limited ethnobotanical documentation on medicinal plants and minimum phytomedicine preparation of crude extracts and isolation of active ingredients [14]. Besides, the rural population of Tigray in general and the people of Ganta Afeshum District in particular greatly depend on medicinal plants because of their acceptability, availability, affordability, and efficacy to treat human and livestock health problem and due to lack of certain infrastructure like roads, ambulance, hospital, and health center. However, these important medicinal plants become exhausted mainly due to agricultural and urban expansion as well as deforestation and heavy livestock grazing pressure.

Available reports show that limited ethnobotanical studies have been conducted in Tigray to document the use of medicinal plants [14–20]. The studies conducted in the districts of Alamata [14], Enderta [15], Hawzen [16], LaelayAdi-yabo [17], Asgede Tsimbela [18], Ofla [19], and Kilte Awulaelo [20] documented 25, 27, 33, 37, 68, 113, and 114 medicinal plants, respectively. However, no such study has so far been conducted in Ganta Afeshum District. Therefore, the protection of these resources and documentation of related traditional knowledge are needed, and it is on the basis of this gap that the present study was undertaken. The study examined and documented the diverse medicinal plant species which are used by the people of Ganta Afeshum District, Eastern Tigray Regional State Northern Ethiopia to treat different human and livestock ailments.

## Materials and methods

### Description of the study area and selection of study sites

**Fig. 1:**
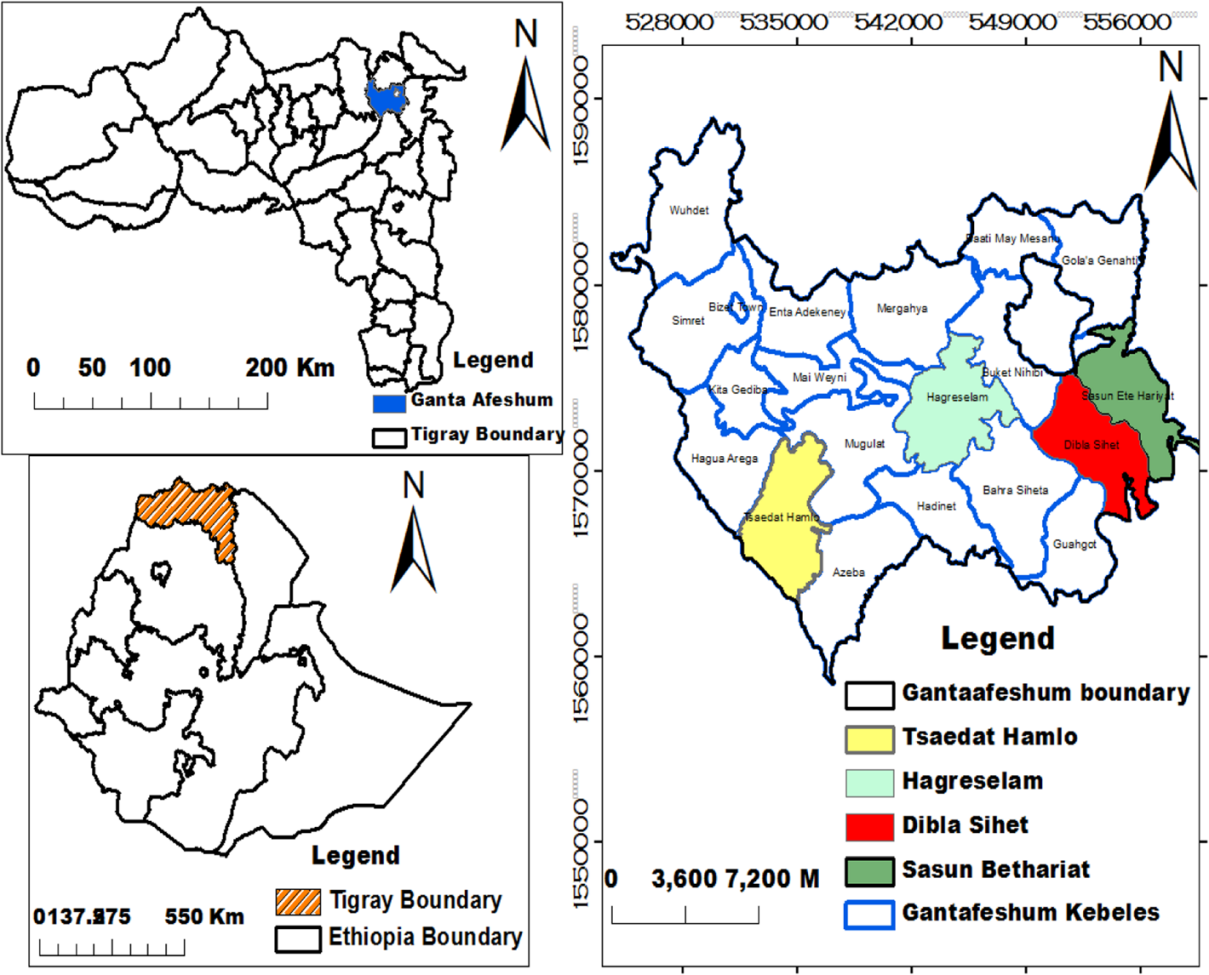
Map of the study area

Tigray is located in Northern Ethiopia at 12° and 15° latitude and 36° and 40° N east longitudes. The total area of Tigray is about 53,000 km^2^ with an average population density of 65/km^2^, and the population growth rate is 3%. Most part of Tigray is arid or semi-arid with annual rainfall of 450 to 980 mm. The total population is about 5.5 million, out of which 85% inhabit rural areas, deriving a livelihood from mixed crop/livestock subsistence agriculture [21]. The study area Ganta Afeshum District lies between 14° 20′ N and 32° 29′ E with a total area of 1636.36 km^2^. It is located 921 km north of Addis Ababa and 120 km north of Mekelle, the capital city of the regional state (Fig. 1).

There are 22,581 households with an average of 4.59 persons for a household in Ganta Afeshum District with a population density of 54.17 persons/km^2^ [21] showing it is one of the most densely populated districts in Tigray Region. The main economy of the population in the wereda has full agriculture based on a mixed farming system. There are only 5 clinics with 114 health servants which provide modern health services. But, these health service buildings and the health servants cannot satisfy the needs of the huge population.

Based on the information gathered by a reconnaissance survey, four kebeles (study sites) at different distance from the administrative center of Adigrat were purposefully selected for the collection of ethnobotanical data. The four selected study sites were Sasun-Bethaweriat, Hagereselam, Dbla-Siet, and Tsaedat-Hamlo. The criteria for the selection of these study sites were availability of traditional practitioner healers and vegetation cover.

### Selection of informants

A total of 78 individuals (54 men and 24 women) were selected randomly and purposefully with different ages (see Additional file 1: Table S1). Out of the 78 individuals, 58 were selected randomly by a lottery method from the total households in order to give equal chances, and 20 key informants who are traditional healers and knowledgeable persons were selected purposefully based on the recommendations of local authorities, elders, and religious leaders. The key informants in the study area are very few and they were purposely selected because of their knowledge and relevance (Table 1).

**Table 1:** Number of general and key informants

District	Kebele	Total households	General informants			Key informants			Total
Ganta Afeshum			M	F	Total	M	F	Total	
	Tsaedat-Hamlo	1082	7	5	12	5	1	6	18
	Hagerselam	1308	13	4	17	3	2	5	22
	Sasun-Bethaweriat	1094	10	4	14	3	2	5	19
	Dbla-Siet	1114	11	4	15	2	2	4	19
Total		4598	41	17	58	13	7	20	78

### Determining sample size

In order to collect ethnobotanical data, men and women household informants with different age were selected from four kebeles, and the sample size was determined using Cochran’s sample size formula as indicated by Bartlett et al. [22] as follows:

$$ n=\frac{N}{1=N{(e)}^2} $$

where *n* is the sample size of the research, *N* is the total number of households in the district (22581), *e* is the maximum variability of making error 5% (0.05), and 1 is the probability of event occurring.

$$ n=22581/1+22581{(0.05)}^2 $$

*n* = 393 which is based on the total number of households of the district (from the 20 kebeles of the district); but our study sites were four kebeles. Therefore, the sample size for each of these “four kebeles” was calculated using the proportion of the number of households in each kebele to the total number of the household in the district.

### Collection of ethnobotanical data

Ethnobotanical data were collected during January and February 2017 through semi-structured interviews, field observation, guided field walk, and focus group discussion. The semi-structured interviews were based on the questions prepared beforehand in English language that were translated into Tigrigna that is the mother language of the informants.

#### Semi-structured interviews

The semi-structured interviews followed Martin [23] in order to obtain ethnobotanical information such as medicinal plant species, common human and livestock ailments, methods of preparation, dosage, routes of administration, vernacular names of the medicinal plants, plant parts used, and conservation and threats of the medicinal plants.

#### Field observation

During the field observations, information about land form, soil type, distribution of medicinal plants, conservation activities and threats of medicinal plants, habit, and habitat of medicinal plants was recorded on site.

#### Guided field walks

Guided field walks were carried out with the assistance of local guides and interviewees on the study sites combined with interviews in order to obtain essential ethnobotanical information as well as to gather medicinal plant specimens by recording all the necessary information of the particular medicinal plant species, such as local name, parts used, and diseases treated by the medicinal plant.

#### Group discussions

Group discussions were made with seven to ten informants at each study site composed of knowledgeable traditional healers in order to collect information about the local soil and land classification, topographic classification, indigenous vegetation classification, and threats and conservation activities of medicinal plants.

### Medicinal plant specimen collection and identification

During the field investigation, plants with medicinal value were collected from home gardens and from the wild and cultivated areas. Essential information such as local name and habit was recorded and herbarium specimens collected. For plant identification, the Flora of Ethiopia and Eritrea [24–31] was used. The accuracy of the identifications was confirmed by the comparison with the deposited authenticated specimens from Addis Ababa University Herbarium and by the help of taxonomists.

### Data presentation and analysis

The ethnobotanical data were analyzed both qualitatively and quantitatively using informant consensus factor (ICF), fidelity level index (FLI), preference ranking, paired comparisons, Jaccard’s coefficient of similarity, and direct matrix ranking. Diseases recorded in this study were grouped into nine major categories associated with specific symptoms and signs with the help of a medical doctor, and informant consensus factor (ICF) was calculated to determine the effectiveness of medicinal plants in each ailment category according to Heinrich et al. [32]. The ICF computed every category to discover the accord of informants on the reported therapy for the group of diseases. It was calculated as follows: *numbers of use citation in each category (n*_*ur*_*) minus the number of species used (n*_*t*_*), divided by the number of use citations in each category minus one.* The result of the calculation (ICF) is from 0 to1. According to Heinrich et al. [32], the higher the value, the more consensuses of the informants.

$$ \mathrm{ICF}={n}_{ur}-{n}_t $$

$$ {n}_{ur-1} $$

where ICF is the informant consensus factor, *n*_*ur*_ is the number of use citation in each category, and *n*_*t*_ is the number of species used.

The FL index quantifies the importance of a species for a given purpose. Most commonly used medicinal plants have high fidelity level index, thus used and agreed by large number of people, whereas medicinal plants that are not commonly used have low fidelity level index and the informants vary on that species in the treatment of particular ailments [33]. Fidelity level index was used to determine the relative healing potential of medicinal plants against human or livestock ailments based on the proportion of informants’ agreement on the use of a given medicinal plant. The formula for FL is given as [34]:

$$ \mathrm{FL}\%=\mathrm{Ip}/\mathrm{IU}\times 100 $$

where FL% is the percentage of fidelity level, Ip is the number of informants who independently indicated the use of a species for the same major ailments, and IU is the total number of informants who mentioned the plant for any major ailment.

The use value was also calculated to see the relative importance of selected traditional medicinal plant species for treating diseases in the study area according to Phillips et al. [35]. It was calculated by the formula UV = Σ*Ui*/*n* where UV stands for the total use value of the traditional medicinal plant species, *U* refers to the number of use reports cited by each informant for a given plant species, and *n* stands for the total number of informants interviewed for a given plant species.

Preference ranking was conducted by asking informants to rank the most important medicinal plants that were frequently used by the local community based on their preference and the importance in the community. The most preferred medicinal plants scored 5 while the least preferred medicinal plant by the informants scored 1. These numbers were summed for all informants, giving an overall ranking for the medicinal plants by sample group of the informants [23].

Direct matrix ranking draws explicitly upon multipurpose dimensions. Direct matrix ranking was performed following the method of Martin [23] to medicinal plant species for their multipurpose use and to relate this to the extent of its utilization versus its dominance. The values of each use diversity for a species were taken, and the value of each species was summed and ranked.

A paired comparison was conducted following [23]. A list of the pairs of selected medicinal plants with all possible combinations was made, and a sequence of the pairs and the order within each pair were randomized before every pair was presented to selected informants; their response recorded and the total value summarized. Besides, independent sample *t* test was calculated in order to compare the average traditional medicinal plant knowledge of men and women informants by using SPSS software.

Jaccard^**’**^s coefficient of similarity (JCS) was performed to evaluate medicinal plant species composition and similarity among different areas. The similarity was calculated between the present study area (Ganta Afeshum District) and other areas of a similar agroecological zone which have been studied by other researchers in different parts of Ethiopia. The formula of JCS is represented as [36]:

$$ \mathrm{JCS}=\frac{c}{\left(a+b+c\right)} $$

where JCS is Jaccard’s coefficient of similarity, *a* is the number of species which is found in habitat A, *b* is the number of species found only in habitat B, and *c* is the number of common species found in habitats A and B.

## Results

The informants involved in the present study were 17–79 years old with an average age of 47 years. From the total informants, 45 (57.7%) were in the age range of 38–58, while 17 of the informants were 59–79 years old and 16 were in the age range of 17–37 years old (see Additional file 1: Table S1).

More than half of the informants (43, 55.1%) were illiterate, and 21 (26.6%) of the informants had been in school for 1–8 years, 12 (15%) of the informants finished school in grade 9–12, and the remaining 2 had schooling above grade 12. From the 54 men informants, 47 were married while 7 men informants were single. From the total of 24 women informants, 11 were married whereas 13 women informants were single.

### Medicinal plants in the study area

#### Diversity of medicinal plants

From the four study sites, a total of 173 medicinal plant species were documented (see Additional file 2: Table S2). These were distributed across 77 plant families and 156 genera. The family Fabaceae stood first by contributing 17 (9.8%) species followed by Lamiaceae and Solanaceae with 9 (5.2%) species each (Table 2).

**Table 2:** Diversity of medicinal plant species belonging to each plant family

No.	Family	Number of medicinal plant species	Percentage
1	Fabaceae	17	9.8
2	Lamiaceae	9	5.2
3	Solanaceae	9	5.2
4	Asteraceae	8	4.6
5	Apiaceae	6	3.5
6	Euphorbiaceae	6	3.5
7	Asclepiadaceae	5	2.9
8	Cucurbitaceae	5	2.9
9	Amaranthaceae	4	2.3
10	Moraceae	4	2.3
11	Polygonaceae	4	2.3
12	Rutaceae	4	2.3
	Others	92	53.2
	Total	173	100

#### Distribution of medicinal plants in the study sites

**Fig. 2:**
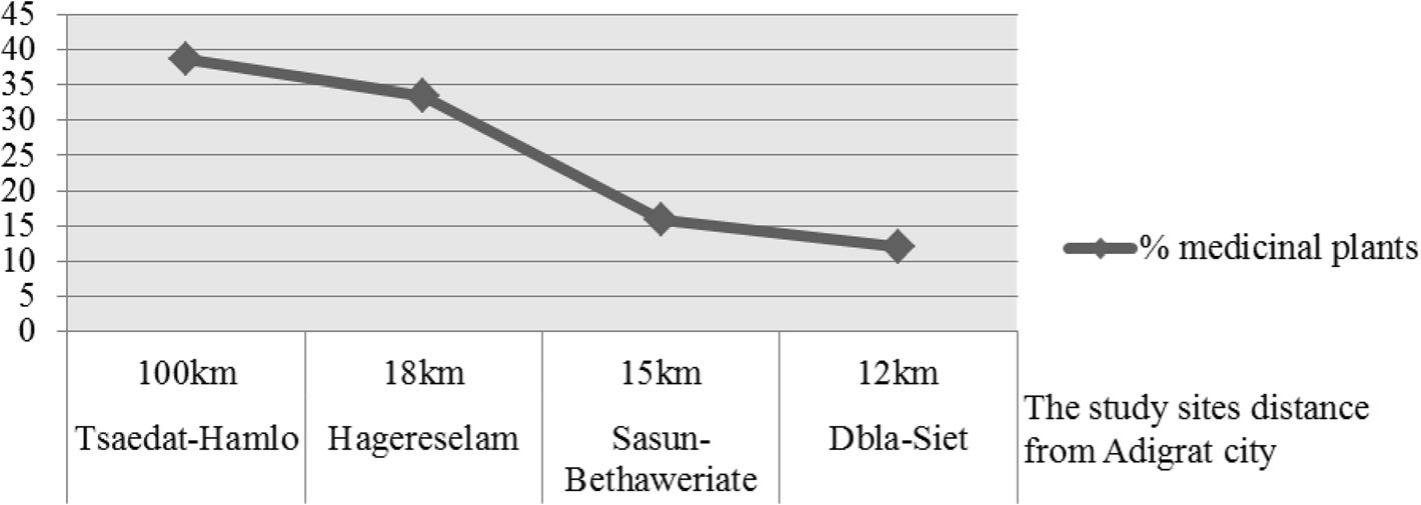
Distribution of medicinal plants in the four study sites

The medicinal plants were unevenly distributed in the four study sites: 38.6% in Tsaedat-Hamlo, 33.5% in Hagereselam, 15.9% in Sasun-Bethaweryat, and 12.1% in Dbla-Siet (Fig. 2).

#### Source of medicinal plants

**Fig. 3:**
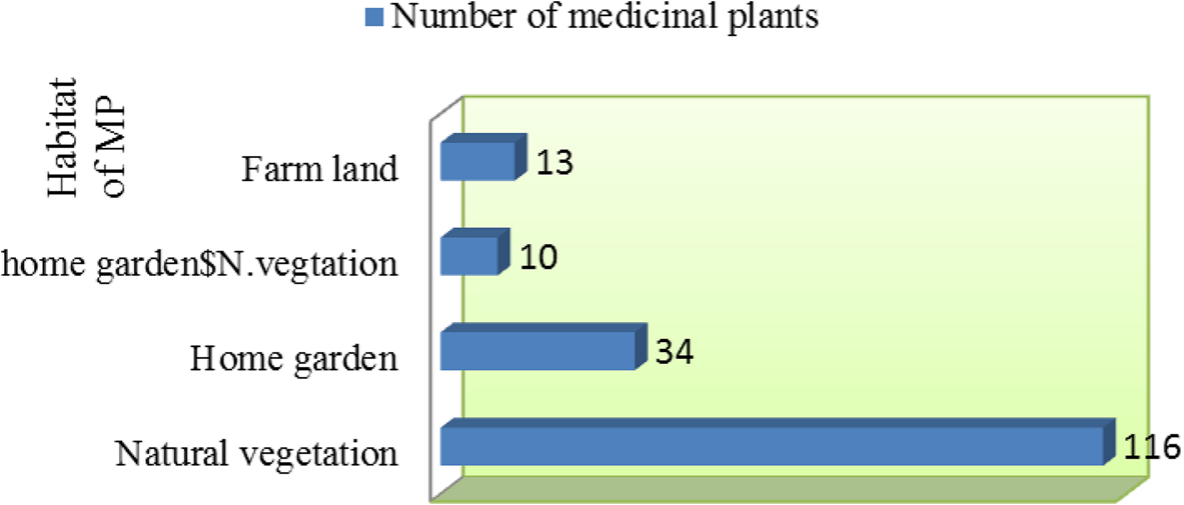
Source of medicinal plants in the study area

From the 173 medicinal plant species, 116 (67.4%) were gathered from the natural vegetation followed by 34 (19.7%) from home gardens (Fig. 3).

#### Growth form (habit) of medicinal plants

**Fig. 4:**
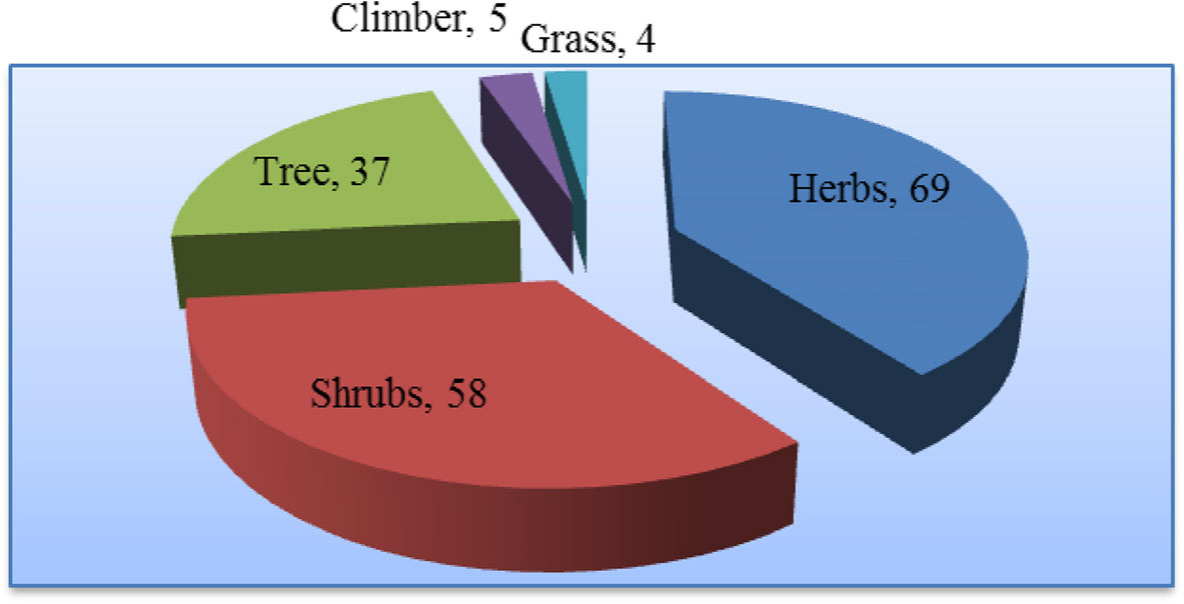
Habit of medicinal plants

The collected medicinal plant species have diverse life forms. From a total of 173 medicinal plants, 69 (39.9%) were herbs which constitute the highest number followed by shrubs 58 (33.5%) (Fig. 4).

#### Parts of the medicinal plants used

Leaves were the most commonly used part of the medicinal plants and accounted for 129 species (38.6%) followed by roots 57 (17.4%) and seeds 38 (11.4%) (Table 3).

**Table 3:** Medicinal plant parts used in traditional medicines

Part used	Number	Percentage
Leaf	129	38.62
Root	57	17.06
Seed	38	11.38
Fruit	32	9.58
Bulb	23	6.88
Bark	19	5.68
Latex	11	3.29
Stem	7	2.09
Leaf and root	6	1.79
Whole plant	5	1.49
Flower	3	0.89
Leaf and stem	2	0.59
Root and bark	2	0.59
Total	334	100

### Conditions of preparation

**Fig. 5:**
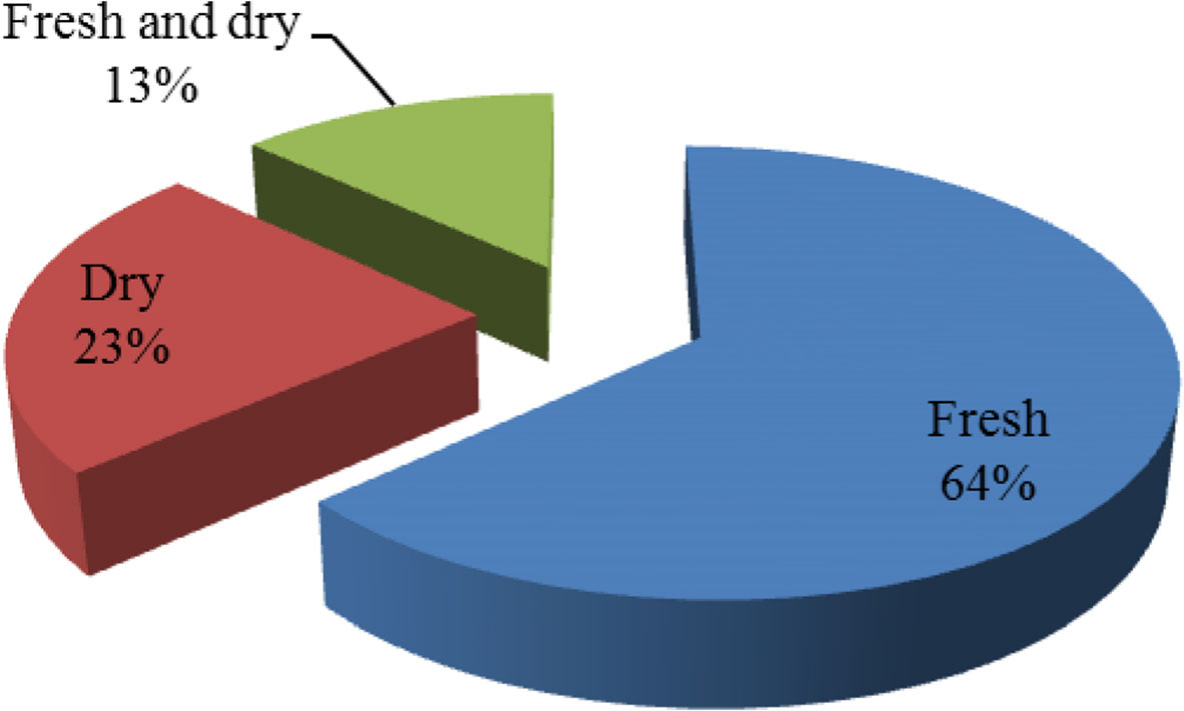
Condition of remedy preparation of medicinal plants

Plants were prepared fresh, dry, or both fresh and dry. The majority of 212 (64%) were prepared in fresh form followed by dry 78 (23%) (Fig. 5).

### Method of preparation

It is known that there are different ways to prepare medicinal plants to treat human and livestock ailments. In the case of Ganta Afeshum District, the major method of preparation was direct and immediate/unprocessed use of the medicinal plants which amounted to 17.9%, followed by grinding 16.8% (Table 4).

**Table 4:** Methods used in the preparation of remedies

No.	Method of preparation	Frequency	Percentage
1	Direct and immediate/unprocessed use of medicinal plants	60	17.96
2	Grinding part of the medicinal plants	56	16.75
3	Grinding then mixing with water, honey, and other	52	15.57
4	Boiling in water, milk, honey, and other	37	11.08
5	Grinding and then filtering	28	8.38
6	Powdering and then mixing with water, honey, and other	27	8.08
7	Burning	17	5.09
8	Powdering	16	4.79
9	Heating	7	2.09
10	Squeezing	6	1.79
11	Roasting	4	1.2
12	Powdering and cooking	3	0.9
13	Grinding and boiling	4	1.2
14	Grinding and soaking	2	0.6
15	Grinding and then burning	2	0.6
16	Powdering and heating	2	0.6
17	Grinding and squeezing	2	0.6
18	Powdering and boiling	2	0.6
19	Powdering and smoking by burning	2	0.6
20	Powdering and heating then mixing	2	0.6
21	Roasting and then grinding	2	0.6
22	Soaking	1	0.3
	Total	334	100

#### Routes of administration

**Fig. 6:**
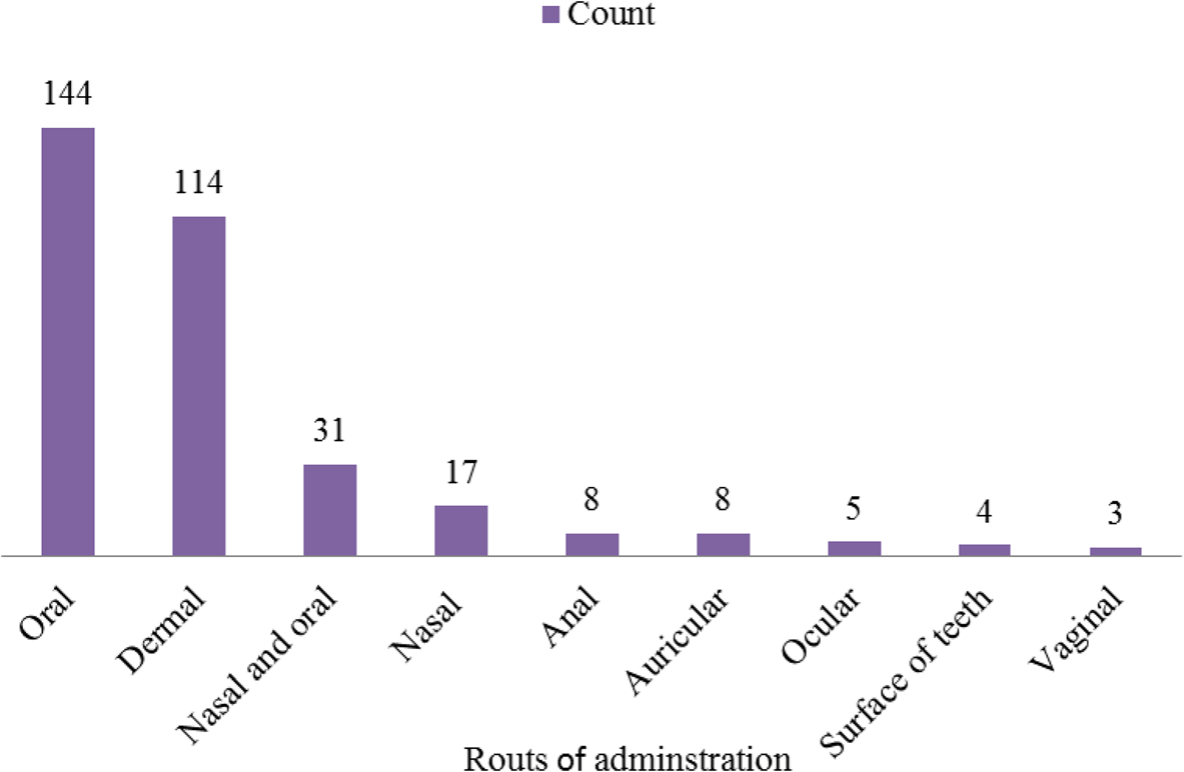
Route of remedy administration for treatment of human and livestock ailments

The result showed that the traditional medicine was administered through different routes; the most common one was orally that accounted for 144 (43.1%) followed by dermal which account for 114 (34.1%) (Fig. 6)**.**

### Modes of application

The data collected from the study area showed that many of the prepared remedies were taken by drinking that accounted for 24.3% followed by smearing 22.8% (Table 5).

**Table 5:** Methods of application of medicinal plants

No.	Modes of application	Frequency	Percentage
1	Drinking	81	24.25
2	Smearing	76	22.75
3	Eating	35	10.48
4	Fumigation	34	10.18
5	Chewing	19	5.69
6	Tie	17	5.09
7	Swallowing	15	4.49
8	Sniffing	13	3.89
9	Rubbing	13	3.89
10	Dropping	12	3.59
11	Washing	8	2.39
12	Smelling	3	0.89
	Total	334	100

### Solvents and ingredients used

The preparation of traditional medicine needs solvents and ingredient. The major solvent was water that accounts for 34.4%, but honey, butter, and cereal oils were also widely used ingredients (Table 6).

**Table 6:** Solvents and ingredients used in the preparation of traditional medicines

No.	Solvents and ingredients	Frequency	Percentage
1	Water	43	34.4
2	Honey	28	22.4
3	Butter	19	15.2
4	Cereal oils	7	5.6
5	Tella/teji/brzi	6	4.8
6	Milk/ergo	5	4
7	Tea/coffee	4	3.2
8	Animal fat	4	3.2
9	Animal urine	1	0.8
10	Animal bile	1	0.8
11	others	7	5.6
	Total	125	100

#### Ailments of humans that can be treated by medicinal plants

In the study area, 74 human ailments were identified to be treated by many medicinal plants (see Additional file 3: Table S3). It was found that single medicinal plant species can treat a number of human ailments, and single ailments can be treated by many medicinal plant species. For example, wounds can be treated by 20 medicinal plants and febrile illness, abdominal pain, headache, and cough can be treated by 15 medicinal plant species each (Table 7).

**Table 7:** Human ailments that can be treated by medicinal plants

No.	Human ailments	No. of medicinal plants used to treat the ailment
1	Wound	20
2	Febrile illness	15
3	Abdominal pain	15
4	Headache	15
5	Cough	15
6	Evil eye	12
7	Evil spirit	11
8	Men impotence	9
9	Tonsillitis	8
10	Bone dislocated	8
11	Hemorrhoids	8
12	Ear infection	8
13	Asthma	8
14	Skin rash	7
15	Toothache	6
16	Tapeworm	6
17	Constipation	6
18	Cutaneous leishmaniasis	6
19	Body swelling	5
20	Paralysis	5
	Other	118
	Total	311

### Ailments of livestock that can be treated by medicinal plants

In the study area, 96 medicinal plants were identified for the treatment of 23 livestock ailments (see Additional file 4: Table S4). Like for humans, one livestock ailment can be treated by several medicinal plants; for instance, leech can be treated by 12 medicinal plants, diarrhea and shivering (locally called halfyen) can be treated by 13 medicinal plants each (Table 8).

**Table 8:** Livestock ailments that can be treated by medicinal plants

No.	Livestock ailment	No. of medicinal plants used to treat livestock ailment
1	Leech	12
2	Diarrhea and shivering	13
3	Bloating	11
4	Newcastle disease	7
5	Abdominal pain	7
6	Wound	5
7	Body swelling	5
8	Hornworm	4
9	Evil spirit	4
10	Bone fracture	4
11	Fleas and lice	3
12	Eye diseases	3
13	Anthrax	3
14	Ticks	3
15	Skin rash	2
16	Prolonged delivery	2
17	Fascioliasis	2
18	Blackleg	1
19	Urine retention	1
20	Rabies	1
21	Malaria	1
22	Cough	1
23	Evil eye	1
	Total	96

### Medicinal plants used for treatment of both humans and animals

In Ganta Afeshum District, 15 types of human and livestock ailments were recorded and 22 medicinal plants were identified to treat both human and livestock ailments (Table 9) (see Additional file 5: Table S5).

**Table 9:** Human and livestock ailment that can be treated by medicinal plants

No.	Human and livestock ailment	No. of medicinal plants used to treat human and livestock ailment
1	Abdominal pain	4
2	Diarrhea	2
3	Bone fracture	2
4	Malaria	2
5	Skin rash	2
6	Wound	1
7	Cough	1
8	Evil spirit	1
9	Evil eye	1
10	Eye diseases	1
11	Urine retention	1
12	Body swelling	1
13	Prolonged delivery	1
14	Rabies	1
15	Dislocated bone	1
	Total	22

## Informants’ knowledge on traditional medicinal plants

### Comparison between sexes

The result for the comparison between men and women in traditional medicinal plant knowledge showed that the difference is not statistically different (Table 10).

**Table 10:** Independent sample *t* test to compare men and women knowledge of traditional medicinal plants

Social group	Informants type	*N*	Average	SD	*t* value	df	*p* value
Gender	Men	54	11.70	7.830	− 0.795	76	0.429
	Women	24	13.21	7.431	− 0.812	46.4	0.421

### Comparison between married and single informants

The result of independent sample *t* test indicated that there is a significant knowledge difference between married and single informants (Table 11).

**Table 11:** Traditional medicinal plant knowledge of married and single informants

Parameter	Group of informants	*N*	Mean	Std. deviation	*t* value	df	*p* value
Marital status	Married	58	12.64	7.357	2.738	76	0.008
	Single	20	7.65	5.923	3.043	40.75	0.004

### Comparison between the key and general informants

Analysis using the SPSS computer program showed that there was a significant mean knowledge difference between the key informants and general informants (Table 12).

**Table 12:** Traditional medicinal plant knowledge of key and general informants

Parameter	Category of informants	*N*	Mean	Std. deviation	*t* value	df	*p* value
Way of selection	General informants	58	9.6724	5.52947	5.827	76	.000
	Key informants	20	19.4000	8.60477	4.730	24.630	.000

### Differences in knowledge depending on educational background

There was a significant negative correlation between the informants’ educational level and the number of medicinal plants reported (Pearson correlation coefficient, *r* = − 0.959, at *α* = 0.05, *p* = 0.041).

### Differences in knowledge depending on age

There was a positive correlation between the age and the knowledge of traditional medicinal plants of the informants, in the study area (Pearson correlation coefficient, *r* = 0.339, *p* = 0.780).

### Informant consensus factor

The informant consensus factor (ICF) was calculated. The highest values were obtained for febrile illness and tonsillitis (0.866) followed by abdominal pain, diarrhea, tapeworm, amoeba and gastritis (0.645), and wound, skin rash, cutaneous leishmaniasis, ringworm, irritation, and skin rash (0.458). Ear infection, eye problem, and the category of heart diseases, blood pressure, and Rh factor had lower ICF (Table 13).

**Table 13:** Informant consensus factor for categorized diseases

No.	Diseases category	Nur	Nt	ICF
1	Skin problems such as wound, skin rash, cutaneous leishmaniasis, ringworm, irritation, and skin rash	73	40	0.458
2	Gastrointestinal problems such as abdominal pain, diarrhea, tapeworm, amoeba, and gastritis	94	34	0.645
3	Evil eye, evil spirit, sray/dgam	43	28	0.357
4	Febrile illness, tonsillitis	168	25	0.866
5	Ear infection, eye diseases	19	14	0.277
6	Malaria, snake bite, rabies, scorpion bite	12	8	0.363
7	Men impotence, abortion, fear and dislike of sex in women	21	14	0.35
8	Headache, toothache, dandruff	38	22	0.432
9	Heart disease, blood pressure, Rh factor	7	6	0.166

#### Fidelity level index

*Withania somnifera*, *Lagenaria siceraria*, *Nigella sativa*, *Laggera tomentosa*, *Silybum marianum*, *Plectranthus lanuginosus*, *Linum usitatissimum*, *Chenopodium ambrosioides*, *Vernonia amygdalina*, and *Asparagus africanus* had the highest fidelity level values, and this was an indication of their good healing potential in the study area (Table 14).

**Table 14:** The relative healing potential of 15 most cited medicinal plants used against human ailments

No.	Scientific name of the plant	Examples of ailment treated	Ip	Iu	FL%
1	*Withania somnifera*	Febrile illness	12	12	100
2	*Lagenaria siceraria*	Wound	2	2	100
3	*Nigella sativa*	Abdominal pain	1	1	100
4	*Laggera tomentosa*	Bleeding	5	5	100
5	*Silybum marianum*	Impotence in men	1	1	100
6	*Plectranthus lanuginosus*	Tonsillitis	1	1	100
7	*Linum usitatissimum*	Constipation	3	3	100
8	*Chenopodium ambrosioides*	Snake bite	1	1	100
9	*Vernonia amygdalina*	Fungal infection	7	7	100
10	*Asparagus africanus*	Evil eye	2	2	100
11	*Citrus limon*	Skin problem	23	24	95.83
12	*Ruta chalepensis*	Cough	19	20	95
13	*Acokanthera schimperi*	Hemorrhoids	11	12	91.66
14	*Euclea racemosa*	Toothache	7	8	87.5
15	*Aloe megalacantha*	malaria	9	11	81

*FL%* percentage of fidelity level, *Ip* the number of informants who independently indicated the use of a species for the same major ailments, *Iu* the total number of informants who mentioned the plant for any major ailment

#### Preference ranking

The five most mentioned medicinal plants (Table 15) were reported for the efficient treatment of febrile illness, and they were selected for preference ranking. Ten key informants were asked to rank the given medicinal plants based on their usefulness, 5 for the medicinal plant which they thought is the most successful for the treatment of febrile illness, and 1 for the least effective plant. *Cordia africana* was ranked first (Table 15).

**Table 15:** Preference ranking of medicinal plants used for the treatment of febrile illness

Scientific name of medicinal plant	Informants (1–10)										Total	Rank
	1	2	3	4	5	6	7	8	9	10		
*Cordia africana*	5	5	4	5	5	4	5	5	5	5	48	1st
*Laggera tomentosa*	3	4	1	3	2	4	3	2	1	4	27	4th
*Medicago polymorpha*	5	1	3	4	5	4	3	3	4	1	33	3rd
*Schinus molle*	2	1	3	1	3	3	2	3	2	1	21	5th
*Vernonia amygdalina*	4	5	3	4	3	5	4	2	3	4	37	2nd

#### Use value and use diversity of medicinal plants in the study area

Of the total 173 medicinal plants documented, 50 (28.90%) had only medicinal importance. The other 123 (71.09%) species had some additional purpose besides medicinal value (Table 16).

**Table 16:** Use diversity of medicinal plants in the study area

Uses	No. of species	Percentage
Only medicinal role	50	28.90
Medicinal plus other uses	123	71.09
Edible	20	11.56
Forage	10	5.78
Washing “soap/detergent”	5	2.89
Tooth brush	15	8.67
Spices	7	4.04
House construction	13	7.51
Fence	14	8.09
Stick	6	3.46
Fuelwood	9	5.20
Shade	11	6.35
Local alcoholic preparation	8	4.62
Glue	5	2.89

The calculated results of use values (UV) showed that *Rhamnus prinoides* scored the highest use values (4.5) followed by *Cordia africana* and *Ruta chalepensis* than other species (Table 17).

**Table 17:** Use value of the most important medicinal plant species in the study area

Scientific name of medicinal plant	∑*Ui*	*n*	UV
*Rhamnus prinoides*	18	4	4.5
*Cordia africana*	60	15	4.00
*Ruta chalepensis*	15	4	3.75
*Allium sativum*	11	3	3.66
*Schinus molle*	25	7	3.57
*Vernonia amygdalina*	31	9	3.44
*Lepidium sativum*	85	25	3.40
*Withania somnifera*	40	12	3.33
*Olea europaea*	13	4	3.25
*Acacia albida*	6	2	3.00

#### Direct matrix ranking

In addition to medicine, the local community used the plants for various purposes such as firewood, charcoal making, for eating as edible fruit, construction, and furniture. The result of direct matrix ranking showed that *Carissa spinarum*, *Acacia etbaica*, *Juniperus procera*, *Cordia africana*, *Olea europaea*, *Mimusops kummel*, *Ziziphus spina-christi*, and *Acacia albida* were ranked first to eighth, respectively. Likewise, the six use values report on eight selected plant species were summed up and ranked, and the result showed firewood, charcoal, medicinal, construction, furniture and farm tools, edible fruit were ranked first, second, third, fourth, fifth, and sixth, respectively (Table 18).

**Table 18:** Direct matrix ranking of eight plant species by four informants based on six use criteria (5 = best; 4 = very good; 3 = good; 2 = less used; 1 = least used, and 0 = no value)

Plant species	Use categories																								Total	Rank
	Medicinal				Furniture and farm tools			Construction					Edible fruit				Charcoal				Firewood					
	Informants (I_1_–I_4_)				Informants (I_1_–I_4_)			Informants (I_1_–I_4_)					Informants (I_1_–I_4_)				Informants (I_1_–I_4_)				Informants (I_1_–I_4_)					
	1	2	3	4	1	2	3	4	1	2	3	4	1	2	3	4	1	2	3	4	1	2	3	4		
*Carissa spinarum*	5	5	4	5	5	5	5	4	5	5	5	5	5	5	4	5	4	5	5	4	5	5	5	5	115	1st
*Cordia africana*	4	1	3	5	2	3	3	4	3	4	5	2	5	2	3	4	1	2	3	3	4	4	3	4	77	4th
*Olea europaea*	2	2	3	3	3	3	3	3	2	1	4	4	0	0	0	0	4	5	4	5	4	3	4	3	65	5th
*Ziziphus spina-christi*	2	1	3	2	1	2	1	4	2	2	1	3	2	3	3	1	1	2	3	2	1	4	2	3	51	7th
*Mimusops kummel*	2	2	4	3	1	1	3	3	2	1	1	5	2	2	3	3	2	2	2	4	2	2	3	3	58	6th
*Acacia albida*	2	2	3	1	1	3	2	2	1	1	2	2	0	0	0	0	2	2	3	3	2	2	2	2	40	8th
*Acacia etbaica*	5	5	5	5	4	5	5	4	5	5	5	4	0	0	0	0	5	5	5	5	5	5	5	5	97	2nd
*Juniperus procera*	4	4	5	4	3	3	4	3	5	3	4	2	0	0	0	0	5	5	4	4	5	5	5	5	82	3rd
Total	106				98				101				52				111				117					
Rank	3rd				5th				4th				6th				2nd				1st					

#### Paired comparison

The disease tonsillitis, locally known as *hanate* commonly attacks children, and it can be treated by using several medicinal plants. The result indicated that *Rhamnus prinoides* and *Achyranthes aspera* were the most preferred and effective treatment (Table 19).

**Table 19:** Paired comparison of five medicinal plants for treating tonsillitis

Scientific name of medicinal plants	Informants (I_1_–I_10_)										Total	Rank
	I_1_	I_2_	I_3_	I_4_	I_5_	I_6_	I_7_	I_8_	I_9_	I_10_		
*Rumex nepalensis*	0	3	2	4	3	4	2	3	1	4	26	4th
*Buddleja polystachya*	1	2	3	4	3	0	2	3	4	4	30	3rd
*Achyranthes aspera*	3	4	3	3	4	3	4	3	3	4	34	2nd
*Lycopersicon esculentum*	1	3	0	2	1	3	3	4	1	2	21	5th
*Rhamnus prinoides*	4	4	4	3	4	3	4	4	4	4	38	1st

#### Comparison with other districts through Jaccard’s coefficient of similarity

The highest Jaccard’s coefficient of similarity in the composition of medicinal plants was found between the study area and Kilte Awulaelo District, whereas similarity was less with Tahitay Adiyabo and Kafta Humera districts (Table 20).

**Table 20:** Jaccard’s coefficient of similarity (JCS)

Study area and references	*a*	*b*	*c*	JCS	Percentage
Ganta Afeshum District (the study area)	173	–	–		
Ofla District, Ethiopia [19]	120	60	53	0.22	22
Kilte Awulaelo District, Ethiopia [20]	116	57	57	0.278	28
Tahitay Adiyabo and Kafta Humera districts, Ethiopia [46]	131	73	42	0.171	17
Asgede Tsimbila District, Ethiopia [18]	126	21	47	0.24	24

*a* number of species found only in Ganta Afeshum District, *b* number of species found only in other district, *c* number of species found in both Ganta Afeshum District and other district

The degree of similarity between the study area and other areas might relate to vegetation types as well as soil types and climatic conditions in the region.

#### Source and transfer of traditional medicinal plant knowledge

**Fig. 7:**
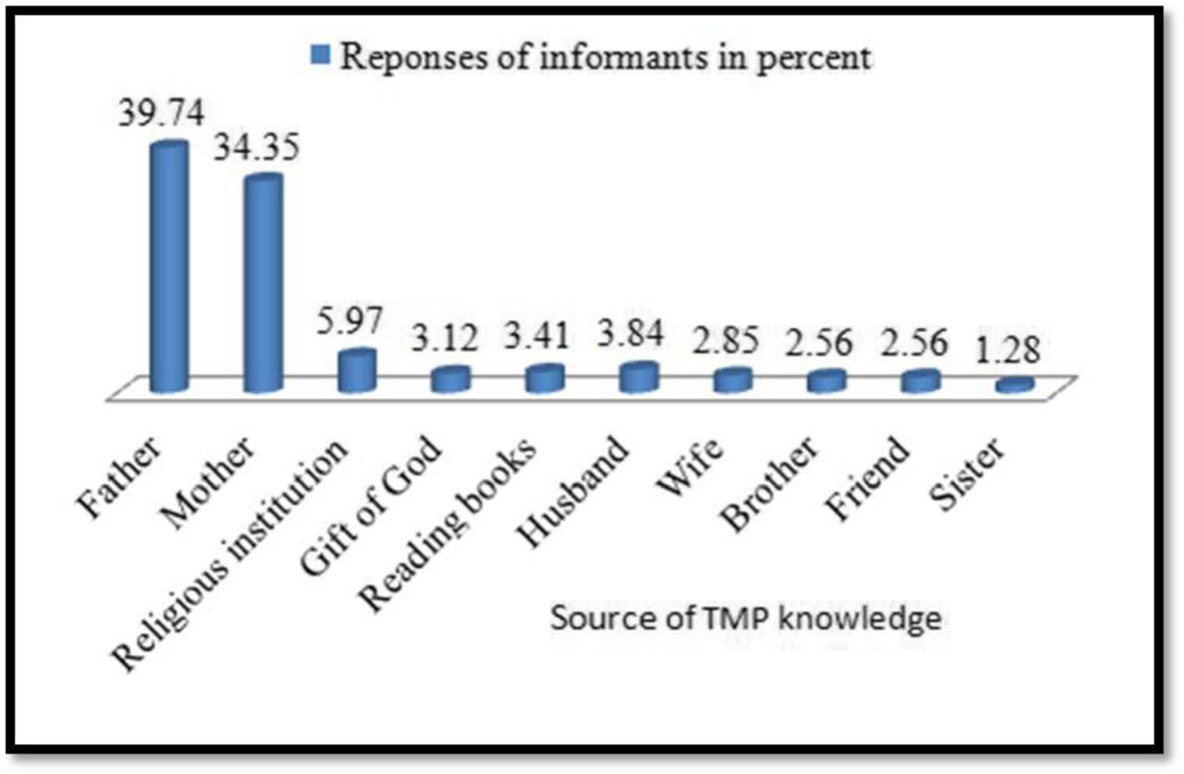
Source of traditional medicinal plant knowledge in Ganta Afeshum District

The highest traditional medicinal plant knowledge was acquired from family members that is 39.74% from the father and 24.35% from the mother, followed by religious institutions (8.9%), reading books (6.41%), and as a gift from God (5.12%) (Fig. 7).

#### Threats to medicinal plants and associated knowledge

**Fig. 8:**
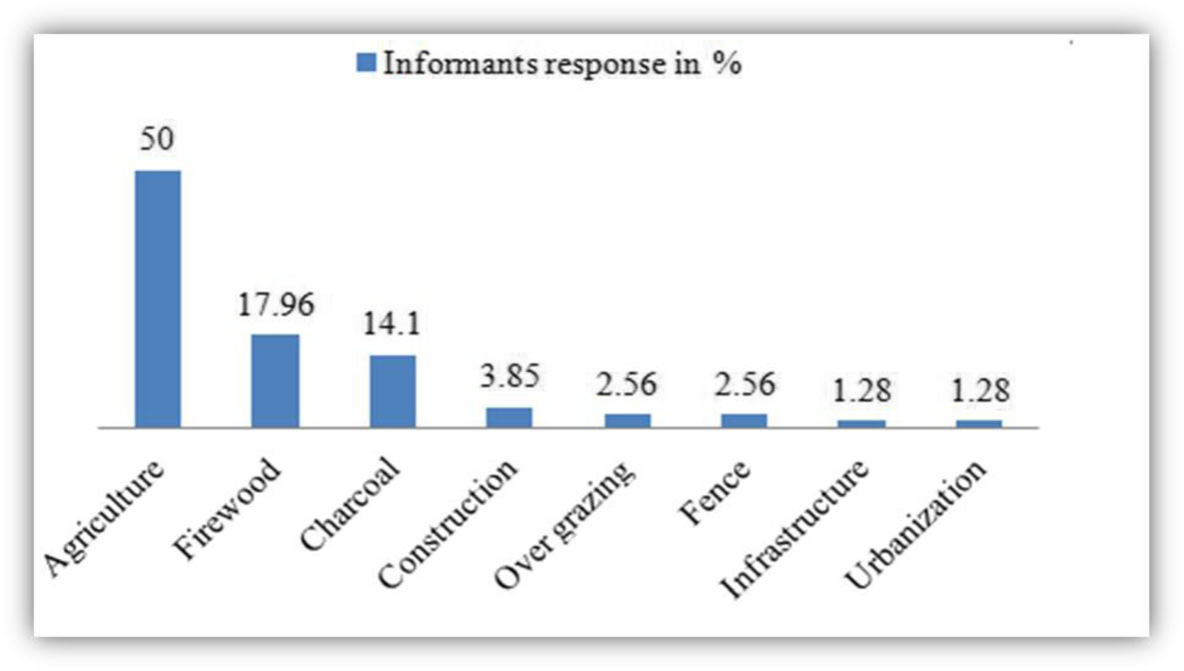
Threats of medicinal plants in the study area

Agricultural expansion was mentioned as the main threat to medicinal plants in the study area followed by cutting trees for firewood and for charcoal making (Fig. 8).

According to the informants, the indigenous knowledge of medicinal plants was faced with many challenges, and the transmission of this knowledge and practice of traditional medicine was in danger due to the unwillingness of the young generation to gain the traditional medicinal plant knowledge. Also, the traditional healers do not show the medicinal plants freely to anybody (Table 21).

**Table 21:** Priority ranking of threats to the knowledge of medicinal plants (values: 1 = least threat, 5 = highest threat)

Threats to MPs knowledge	Informants										Total	Rank
	I_1_	I_2_	I_3_	I_4_	I_5_	I_6_	I_7_	I_8_	I_9_	I_10_		
The traditional healers do not show the medicinal plants	3	5	4	4	3	5	4	4	4	3	39	2nd
Expansion of schools	3	4	3	4	3	4	3	4	3	3	34	3rd
The establishment of health center and posts	3	3	3	3	3	3	3	3	2	3	29	4th
Unwillingness of young generation	5	4	5	5	4	4	4	5	4	5	45	1st
Youth moving to urban areas	3	2	2	3	3	2	2	3	2	3	25	5th

## Discussion

One hundred seventy-three medicinal plant species were identified for the treatment of human and livestock ailments that distributed across 77 families and 156 genera. From the 77 plant families, Fabaceae stood first by contributing 17 (9.82%) species followed by Lamiaceae and Solanaceae that contain 9 (5.2%) species. Similarly, various studies in Ethiopia [2, 11, 37, 38] showed that Fabaceae was the dominant family among the others, whereas other studies [19, 39–44] noted that Asteraceae was the dominant one among others.

The result indicates that medicinal plants are unevenly distributed in the four study sites. More of the medicinal plants were found in Tsaedat-Hamlo and Hagereselam due to certain reasons. Tsaedat-Hamlo is a remote part of Ganta Afeshum District about 100 km from Adigrat. This has caused insufficient coverage of modern medicine, unaffordable as well as inadequate health facilities, and medical personnel. Instead, many people there use the accessible, inexpensive, and locally available traditional medicinal plants. The kebeles Hagerselam and Tsaedat-Hamlo also had a better vegetation cover and more traditional healers than Sasun-Bethaweryat and Dbla-Siet. Also, the far remote kebeles were less influenced by modernization and urbanization. Generally, urbanization and modernization negatively affect the knowledge of traditional medicinal plants. There is also a public health concern as modernization alters the practice of traditional medicine. The loss of traditional medicinal plant knowledge of these kebeles’ people alters health care-seeking behavior. The residents of Sasun-Bethaweryat and Dbla-Siet were more educated and engaged in commercial activities; as a result, they were seeking modern medication. Less educated people tend to be less acculturated and know more medicinal plants while educated people tend to be more acculturated, know few medicinal plants, and seek Western medical treatment [45].

From the total of 173 medicinal plant species, 116 (67.44%) plants were gathered from the natural vegetation followed by 34 (19.65%) from home gardens. This indicates that the communities of the study area highly depend on the wild source to obtain the medicinal plants; in other words, the habit of cultivating medicinal plants in home gardens was not much developed. Similar studies conducted elsewhere in different parts of Ethiopia [10, 45–49] also reported that most of the medicinal plants were collected from natural vegetation.

The medicinal plants in the study area had diverse growth form: herbs 69 (39.88%), shrubs 58 (33.52%), trees 37 (21.39), and climbers 5 (2.89). The dominance of herbs and shrubs is in agreement with several studies conducted in Ethiopia [45, 48, 50–52]. In contrast, Lulekal et al. [2] reported from Mana Angetu District, southeastern Ethiopia, that shrubs there made up the highest proportion of the medicinal plants; the finding of Regassa [38] in Hawassa city, southern Ethiopia, showed that the majority of the collected medicinal plants there were trees, followed by shrubs, herbs, and climbers.

The results showed that the local people of the Ganta Afeshum District use different parts of medicinal plants to prepare remedies. Leaves were the most widely used part, which is an important finding because harvesting leaves does not have detrimental effects on the survival of the medicinal plants, whereas harvesting roots and whole plants has a negative impact on the survival. In the same way, several studies [45, 47, 53–56] have revealed that the leaves of the medicinal plants were repeatedly used for the treatment of human and livestock ailments. On the other hand, Mesfin et al. [48] and Assefa and Abebe [57] reported that the roots were a widely utilized medicinal plant part to treat different ailments.

Most of the medicinal plants (212, 64%) were prepared to be used in the fresh form, and this indicates that fresh medicinal plants are much easier and quicker to prepare for remedy than the other forms. Abebe [11], Gebeyehu [42], and Chekole [45] reported similar results.

In Ganta Afeshum District, the common method of traditional medicine preparation is direct and immediate/unprocessed use of the medicinal plants followed by grinding and boiling in water. Elsewhere in Ethiopia, similar findings were reported [11, 37, 39, 47, 57, 58], and grinding, pounding, smoking, squeezing, burning, roasting, and powdering are common the methods of preparations of traditional medicines.

Oral administration was the most common way for traditional medicine followed by dermal, nasal, and anal. This discovery is in line with many findings of researchers [2, 16, 19, 39, 43, 58, 59] who reported that the major way of administration was oral. In contrast, Teklay et al. [20] reported that dermal was a common way of administration. Many of the prepared traditional medicines were taken by drinking followed by smearing, eating, fumigation, and chewing. This finding is concurrent with the discoveries of Gebeyehu [42] who reported that prepared remedies were applied by drinking, dropping, creaming (ointment), eating, inhaling/sniffing, and sucking. Similarly, Tamene [53] revealed that the medicinal plants prepared by traditional healers were applied by different methods such as drinking, painting, chewing, swallowing, put on, smelling, and smoking. In addition, traditional medicines of the study area were prepared with solvents and ingredients, such as water, honey, butter, cereal oils like sesame oil, teji/tella (local beer), milk/ergo (yoghurt), and tea/coffee. A similar study was carried out in Chifra District, Afar Region, Northeastern Ethiopia, by Seifu [50] who reported the Afar people and their traditional healers used solvents and additives like water, honey, sugar, and milk of goat and camels during the preparation of traditional medicines.

In Ganta Afeshum District, 74 human, 23 livestock, and15 both human and livestock ailments were recognized. This indicated that the people of the district were suffering from many ailments as compared to other areas such as in Gimbi District, western Wellega, where Tolasa [58] identified 49 human and 19 livestock ailments. In Minjar-Shenkora District, North Shewa Zone of Amhara Region, Alemayehu [43] reported 45 human ailments; in Seru District, Arsi Zone of Oromia Region, Gebrehiwot [39] reported 53 human and 17 livestock ailments; and in Wondo Genet natural forest and adjacent kebeles, Sidama Zone, SNNP Region, Tamene [53] recognized 40 human and 17 livestock ailments. Because of this burden of health problem, the people of Ganta Afeshum District widely used many medicinal plants, and that is why such a large number of medicinal plants were identified. Moreover, a single human ailment was found to be treated by several medicinal plants. This is in agreement with the findings of different scholars [40, 41, 43] who have reported wounds, headache, febrile illness, evil eye, tonsillitis, evil spirit, hemorrhoids, toothache, earache, and cough to be treated by several medicinal plants.

Men and women informants had equal traditional medicinal plant knowledge in the study area. This result is in line with the findings of Asnake et al. [55] but disagrees with the discoveries of Teklehaymanot and Giday [59] who showed the presence of a significant difference in traditional medicinal plant knowledge between men and women. On the other hand, married informants reported significantly more medicinal plants than single informants. This is because most of the married informants were adults and more experienced with plant contact. They also possess children and livestock, they lead a family, and they are responsible for the family health care and are also the major players in using medicinal plants. Similarly, Beyene [33] reported that married informants had a better knowledge of traditional medicinal plants than single informants.

Key informants cited significantly more medicinal plants than the general informants. This is because the key informants were traditional herbalists with broad, empirical traditional medicinal plant knowledge. They cultivate, collect, process, prepare, administer, and treat patients by using medicinal plants. General informants cited fewer medicinal plants; even though they perform self-medication (homemade remedies), they are not knowledgeable about medicinal plants. Beyene [33] got similar results.

There was a significant negative correlation between the informants’ educational level and their knowledge of traditional medicinal plants. This means that with a higher level of education, the knowledge of traditional medicinal plants decreases. Thus, modern education weakens the traditional medicinal knowledge of the young generation. This discovery agrees with the research carried out in Dire Dawa city, eastern Ethiopia, by Kebede et al. [47].

The more aged informants were, the more they were knowledgeable about traditional medicinal plants. Similar results were reported by Kebede et al. [47], Kefalew et al. [60], and Birhanu [61]. In the exercise of preference ranking, *Cordia africana* scored first rank, and *Vernonia amygdalina*, *Medicago polymorpha*, *Laggera tomentosa*, and *Schinus molle* scored second to the fifth rank, respectively, for the efficient treatment of febrile illness. In a study from Ofla wereda, the southern zone of Tigray Region [19], *Cynoglossum lanceolatum* was ranked first. Similarly, a study conducted by Chekole [45] in Gubalafto District showed that *Cynoglossum coeruleum* and *Ocimum latifolium* were preferred by the community to treat febrile illness. On the other hand, *Momordica foetida* was ranked first as the most effective for the treatment of rabies among Guji agro-pastoralists, Bule Hora District of Borana Zone, Oromia Region [54], and *Nicotiana tabacum* was ranked first for the treatment of snake bite in Gimbi woreda, western Wellega [58].

Furthermore, in direct matrix ranking, *Carissa spinarum*, *Acacia etbaica*, and *Juniperus procera* were ranked first, second, and third, respectively, showing multipurpose roles and the most preferred and extensively exploited by the local community. For this reason, they were the most threatened plant species in the study area and need conservation priority for their sustainability. Conversely, *Cordia africana*, *Olea europaea*, *Mimusops kummel*, *Ziziphus spina-christi*, and *Acacia albida* were the least preferred multipurpose medicinal plants and less threatened since they are not widely exploited by local communities. Similar studies were carried out elsewhere in other parts of Ethiopia like in Goma Wereda, Jima Zone of Oromia Region, Ethiopia, by Etana [40]. He used the method of direct matrix ranking and revealed that *Cordia africana* was the most preferred and first ranked multipurpose plant species. In another study in Seru wereda, Arsi Zone, Oromia Region, Ethiopia, Gebrehiwot [39] indicated that *Acacia abyssinica* was the most preferred multipurpose plant. A related study by Teklay et al. [20] indicated *Cordia africana*, *Eucalyptus globules*, *Opuntia ficus-indica*, and *Dodonia angustifolia* as the most preferred multipurpose plants by the local people in Kilte Awulaelo District which is from the same zone of the study area demonstrating the presence of cultural use difference of community.

In paired comparison, *Rhamnus prinoides* and *Achyranthes aspera* were ranked first and second indicating being the most preferred and effective for treatment of tonsillitis as compared to *Rumex nepalensis* and *Lycopersicon esculentum.* Similarly, Teklay et al. [20] and Chekole [45] showed *Rhamnus prinoides* and *Achyranthes aspera* were used for the same purpose to treat tonsillitis. Moreover, ten medicinal plant species have the highest use values in the study area, indicating that they are more effective to treat ailments. Among the total documented medicinal plant species, *Rhamnus prinoides* followed by *Cordia africana* and *Ruta chalepensis* were used to treat the highest number of diseases. In this sense, a plant with a high use value would theoretically have a correspondingly high cultural consensus [35]. Therefore, to maintain the continuous use of plant resources in the study area, conservation priorities should be given for those multipurpose and more threatened ones.

## Conclusions

Ganta Afeshum District is relatively rich in medicinal plant species. One hundred seventy-three medicinal plant species were collected and identified. These medicinal plants were used by the inhabitants to treat 112 human and livestock ailments. Wounds, febrile illness, abdominal pain, headache, cough, evil eye, evil spirit, men impotence, and tonsillitis were frequently occurring human ailments, whereas, leech, bloating, Newcastle, and bone fracture were common livestock ailments. This indicated that the local community depends on using medicinal plant species and the associated indigenous knowledge to prevent diverse human and livestock ailments, although modern health services are expanding.

In the study area, there was no knowledge difference between men and women informants on traditional medicinal plant knowledge, whereas, educational level and knowledge of medicinal plants of informants were negatively correlated. Thus, the age and medicinal plant knowledge of the informants were positively correlated, by which the younger informants showed less concern in sharing, recording, and examining processes of traditional medication. Greater preference ranking, use value scores, and fidelity level values of the documented medicinal plant species would enable the forthcoming phytochemical and pharmaceutical studies and conservation activities.

Natural vegetation was the main source of medicinal plants in Ganta Afeshum District followed by home gardens and farmlands. But nowadays, deforestation, agricultural expansion, overgrazing, drought, and overexploitation are threatening these plant resources and their habitat. Therefore, people of the study area should apply complementary conservation approaches (in situ and ex situ) for sustainable use of these resources and prevent species extinction.

## Additional files

Additional file 1: **Table S1.** List of informants contacted in the study area. (DOC 134 kb)

Additional file 2: **Table S2.** List of medicinal plants recorded from the study area. (DOC 325 kb)

Additional File 3: **Table S3.** List of medicinal plants used for treating human ailments. (DOC 402 kb)

Additional file 4: **Table S4.** List of medicinal plants used for treating livestock ailments. (DOC 63 kb)

Additional file 5: **Table S5.** List of medicinal plants used for treating human and livestock ailments. (DOC 164 kb)
