# Supplementary material for: Ethnobotanical study of medicinal plants in Ganta Afeshum District, Eastern Zone of Tigray, Northern Ethiopia
Source: J Ethnobiol Ethnomed. 2018 Nov 3;14:64. doi: 10.1186/s13002-018-0266-z (PMC6215673; doi:10.1186/s13002-018-0266-z)
Supplement: Supplementary file 1 — Table S1. List of informants contacted in the study area. (DOC 134 kb) [file 13002_2018_266_MOESM1_ESM.doc]

Table S1. List of informants contacted in the Study Area

(Key; with ●are key Informants, education level is identified as none = Illiterate numbers (1, 2, 3…) indicate grade completed

| **No** | **Name** | **Sex** | **Age** | **Marital status** | **Education Status** | **Tabya** | **Marital status** | **Occupation** |
| --- | --- | --- | --- | --- | --- | --- | --- | --- |
| 1 | Aba Tesfamariam | M | 65 | S | None | Tsaedat-Hamlo | Married | Monk |
| 2 | Abadit Tsegay | W | 40 | S | None | Sasun-Bethaweryat | Single | Farmer |
| 3 | Abeba G/medhin | W | 59 | S | None | Tsaedat-Hamlo | Single | Farmer |
| 4 | Abreh Yhdego● | M | 50 | M | None | Tsaedat-Hamlo | Married | Farmer |
| 5 | Abreha Zgta | M | 79 | M | None | Hagereselam | Single | Farmer |
| 6 | Abrehet Kasa | W | 45 | M | None | Dbla-Siet | Married | Housewife |
| 7 | Amaha G/kidan | M | 49 | M | None | Tsaedat-Hamlo | Married | Farmer |
| 8 | Aman G/kiros | M | 17 | S | 12 | Dbla-Siet | Single | Student |
| 9 | Berh G/mariam● | M | 40 | M | None | Sasun-Bethaweryat | Married | Farmer |
| 10 | Berihu Hadera | M | 49 | M | 8 | Hagereselam | Married | Farmer |
| 11 | Brhane G/yohans | M | 40 | M | 8 | Hagereselam | Married | Farmer |
| 12 | Brhane Kahsay | M | 40 | M | None | Hagereselam | Married | Farmer |
| 13 | Bsheu Berh● | M | 54 | M | 6 | Hagereselam | Married | Farmer |
| 14 | Bsrat G/mikael● | W | 35 | M | None | Hagereselam | Married | Housewife |
| 15 | Bsrat Gidey● | W | 70 | M | None | Hagereselam | Single | Farmer |
| 16 | Daniet G/yohans | W | 25 | S | 10 | Sasun-Bethaweryat | Single | Student |
| 17 | Desta Grmay | W | 45 | S | None | Sasun-Bethaweryat | Single | Farmer |
| 18 | Eqar Kalayu | W | 50 | M | None | Tsaedat-Hamlo | Married | Farmer |
| 19 | Equbay Mezgebe | M | 57 | M | None | Hagereselam | Married | Farmer |
| 20 | Eysus Fkadu● | W | 50 | M | None | Sasun-Bethaweryat | Married | Housewife |
| 21 | Fkre G/fial | M | 35 | M | 10 | Tsaedat-Hamlo | Married | Farmer |
| 22 | Freweni Teklay | W | 17 | S | 12 | Dbla-Siet | Single | Student |
| 23 | Ftaw Aregawi● | W | 48 | S | None | Tsaedat-Hamlo | Married | Farmer |
| 24 | Ftsumbrhan Tekle● | M | 72 | M | None | Tsaedat-Hamlo | Married | Farmer |
| 25 | G/kirose Belay | M | 50 | M | None | Tsaedat-Hamlo | Married | Farmer |
| 26 | G/krstos G/medhin | M | 20 | M | 2 | Tsaedat-Hamlo | Married | Farmer |
| 27 | G/lasea W/anenia● | M | 66 | M | None | Dbla-Siet | Married | Farmer |
| 28 | G/libanos Hailu● | M | 66 | M | None | Sasun-Bethaweryat | Married | Farmer |
| 29 | G/mariam Hailu | M | 58 | M | None | Sasun-Bethaweryat | Married | Farmer |
| 30 | G/slasea Tekulu | M | 48 | M | 5 | Tsaedat-Hamlo | Married | Farmer |
| 31 | G/tsadik Tesfay● | M | 40 | M | 9 | Sasun-Bethaweryat | Married | Farmer |
| 32 | G/yesus Abreha | M | 65 | M | None | Sasun-Bethaweryat | Married | priest |
| 33 | Gdey G/kidan● | W | 58 | S | None | Sasun-Bethaweryat | Single | Farmer |
| 34 | Gebru Atsbeha | M | 65 | M | None | Hagereselam | Married | Farmer |
| 35 | Getachew Tesfay | M | 25 | M | 10 | Sasun-Bethaweryat | Married | Farmer |
| 36 | Hadgu Mehari | M | 43 | M | 8 | Dbla-Siet | Married | Farmer |
| 37 | Hadush Abreha● | M | 79 | M | None | Dbla-Siet | Married | Farmer |
| 38 | Hadush G/tsadik | M | 67 | M | None | Hagereselam | Married | Farmer |
| 39 | Hagos G/her | M | 73 | M | None | Hagereselam | Married | Farmer |
| 40 | Hagos Teklay | M | 59 | M | 6 | Dbla-Siet | Married | Farmer |
| 41 | Hagos W/mariam | M | 49 | M | 4 | Dbla-Siet | Married | Farmer |
| 42 | Hailay Adhanom | M | 42 | M | 8 | Hagereselam | Married | Farmer |
| 43 | Haileslasea Hailu | M | 28 | M | 6 | Sasun-Bethaweryat | Married | Farmer |
| 44 | Hailu Aregawi | M | 60 | M | None | Sasun-Bethaweryat | Married | Farmer |
| 45 | Hailu G/medhin | M | 65 | M | None | Dbla-Siet | Married | Farmer |
| 46 | Hansu G/hiwot | W | 68 | M | None | Sasun-Bethaweryat | Married | Housewife |
| 47 | Hiwan welay | W | 45 | M | None | Hagereselam | Married | Housewife |
| 48 | Hiwot G/her | W | 67 | S | None | Hagereselam | Single | Farmer |
| 49 | Hiwot Hagos | W | 59 | M | None | Dbla-Siet | Married | Housewife |
| 50 | Kahsay Gdey | M | 52 | M | 6 | Dbla-Siet | Married | Farmer |
| 51 | Kahsay Hailu | M | 42 | M | None | Dbla-Siet | Married | Farmer |
| 52 | Kahsay Tsegay | M | 48 | M | None | Sasun-Bethaweryat | Married | Farmer |
| 53 | Kidane Grmay● | M | 50 | M | None | Hagereselam | Married | Farmer |
| 54 | Letbrhan Berh | W | 30 | S | 6 | Hagereselam | Single | Farmer |
| 55 | Mebrahtu Abreha● | M | 42 | M | 7 | Hagereselam | Married | Farmer |
| 56 | Mebrahtu Aregawi | M | 18 | S | 10 | Dbla-Siet | Single | Student |
| 57 | Mebrhit G/medhin | W | 28 | S | 10 | Hagereselam | Single | Farmer |
| 58 | Medhin Abreha* | W | 45 | S | None | Dbla-Siet | Single | Farmer |
| 59 | Medhin G/her | W | 40 | S | None | Tsaedat-Hamlo | Married | Farmer |
| 60 | Medhin Tesfay● | W | 47 | M | None | Dbla-Siet | Married | Housewife |
| 61 | Mehari Teklay | M | 40 | M | None | Sasun-Bethaweryat | Married | Farmer |
| 62 | Million G/medhin | W | 38 | S | >12 | Tsaedat-Hamlo | Single | Farmer |
| 63 | Nguse Hailu | M | 46 | M | None | Sasun-Bethaweryat | Married | Farmer |
| 64 | Solomon G/hiwot | M | 24 | S | >12 | Hagereselam | Single | Teacher |
| 65 | Solomon Muhur● | M | 40 | S | 10 | Tsaedat-Hamlo | Single | Priest |
| 66 | Tadese G/mikael | M | 35 | M | 10 | Dbla-Siet | Married | Farmer |
| 67 | Tadese Grmay | M | 50 | M | 8 | Hagereselam | Married | Farmer |
| 68 | Tekl Hadgu | M | 43 | M | 5 | Dbla-Siet | Married | Farmer |
| 69 | Teklay Aregawi● | M | 45 | M | 5 | Tsaedat-Hamlo | Married | Farmer |
| 70 | Teklay G/medhin | M | 56 | M | 5 | Tsaedat-Hamlo | Married | Farmer |
| 71 | Teklay G/tsadik | M | 50 | M | None | Hagereselam | Married | Farmer |
| 72 | Tewld G/amlak | M | 20 | S | 12 | Sasun-Bethaweryat | Single | Student |
| 73 | Tsega G/krstos | W | 34 | S | 7 | Dbla-Siet | Single | Farmer |
| 74 | Tsehay Hailay | M | 17 | S | 12 | Dbla-Siet | Single | Student |
| 75 | W/gebriel Asgedom | M | 66 | M | None | Sasun-Bethaweryat | Married | priest |
| 76 | Yowhanse weldu | M | 55 | M | 6 | Hagereselam | Married | Farmer |
| 77 | Ytbark Hailu● | M | 38 | M | 8 | Tsaedat-Hamlo | Married | Priest |
| 78 | Zemeda G/kidan | W | 40 | M | 8 | Tsaedat-Hamlo | Married | Farmer |
