# Supplementary material for: Ethnobotanical study of medicinal plants in Ganta Afeshum District, Eastern Zone of Tigray, Northern Ethiopia
Source: J Ethnobiol Ethnomed. 2018 Nov 3;14:64. doi: 10.1186/s13002-018-0266-z (PMC6215673; doi:10.1186/s13002-018-0266-z)
Supplement: Supplementary file 2 — Table S2. List of medicinal plants recorded from the study area. (DOC 325 kb) [file 13002_2018_266_MOESM2_ESM.doc]

**Table S2 List of medicinal plants recorded from the study area.** (NV=Natural vegetation, Hg= Home garden, Fl= farm land)

| **Family** | **Scientific name** | **Local name/Tigrigna** | **Habit** | **Habitat** | **Collection number** |
| --- | --- | --- | --- | --- | --- |
| Acanthaceae | *Achyranthes aspera* L. | Mechelo | Herb | Nv | GG 013 |
|  | *Hypoestes forskaolii* (Vahl) R.Br. | Grbya | Herb | Nv | GG 064 |
|  | *Justicia schimperiana* (Hochst.ex Nees) T. Anders | Shimeza | Shrub | Nv | GG 008 |
| Alliaceae | *Allium cepa* L. | Keyh-shgurti | Herb | Hg | GG 044 |
|  | *Allium sativum* L. | Tsaeda-shgurti | Herb | Hg | GG 160 |
| Aloaceae | *Aloe camperi* Schweinf | Sandaere | Herb | Nv | GG 139 |
|  | *Aloe megalacantha* Baker | Ere | Herb | Nv | GG 080 |
| Amaranthaceae | *Alternanthera nodiflora*  R. Br. | Kodo-Gih/Tetem –gih | Shrub | Nv | GG 032 |
|  | *Amaranthus caudatus* L. | Hamli-adgi | Shrub | Nv | GG 010 |
|  | *Aerva javanica*  (Burm.f.) Schultes | Lge-Dmu | Shrub | Nv | GG 036 |
| Amaryllidaceae | *Crinum ornatum*  (Ait.) Bury | Shgurti-zbe | Herb | Nv | GG 149 |
| Anacardiaceae | *Rhus glutinosa* A. Rich. | Tetael | Tree | Nv | GG 147 |
|  | *Schinus molle* L. | Tkur-berbere | Tree | Nv | GG 143 |
| Apiaceae | *Conium maculatum* L. | Tsakda | Herb | Hg | GG 169 |
|  | *Cuminum cyminum* L*.* | Kemun | Herb | Hg | GG 055 |
|  | *Dacus carota* L. | Carot | Herb | Hg | GG 057 |
|  | *Foeniculum vulgare* L | Shlan | Herb | Nv | GG 156 |
|  | *Heteromorpha arborescens*  (Spreng.) Cham. & Schltdl*.* | Seseg –zbe | Herb | Nv | GG 134 |
|  | *Trachyspermum ammi* L. | Azmud | Herb | Hg | GG 100 |
| Apocynaceae | *Acokanthera schimperi*  (A.DC.) Schweinif | Mebte | Tree | Nv | GG 029 |
|  | *Calotropis procera* (Ait.) Ait.f. | Gindae | Herb | Nv | GG 045 |
|  | *Carissa spinarum* L. | Agam | Tree | Nv | GG 113 |
| Arecaceae | *Borassus aethiopum* Mart. | Sye | Tree | Nv | GG 170 |
| Asclepiadaceae | *Ceropegia convolvuloides* A. Rich. | Merkah | Herb | Nv | GG 120 |
|  | *Ceropegia vignaldiana*  A. Rich. | Mshko | Herb | Nv | GG 132 |
|  | *Dregea abyssinica*  (Hochst.) K. Schum. | Shankuk | Shrub | Nv | GG 129 |
|  | *Gomphocarpus fruticosus* (L.) Ait.f. | Demayto | Herb | Nv | GG 087 |
|  | *Periploca linearifolia* Quart.-Dill. & A. Rich. | Moder | Tree | Nv | GG 133 |
| Asparagaceae | *Agave americana* L. |  |  |  |  |
|  | *Asparagus africanus* L. | Kesta-Ansti | Shrub | Nv | GG 009 |
| Asphodelaceae | *Kniphofia isoettfolia*  Steud. ex Hochst*.* | Ashenda | Herb | Nv | GG 103 |
| [Asteraceae](https://en.wikipedia.org/wiki/Asteraceae) | *Artemisia abyssinica*  Sch.Bip. ex A.Rich. | Chena wedwado | Herb | Nv | GG 001 |
|  | *Bidens macroptera*  (Sch. Bip. ex Chiov.) Mesfin | Gelgele-meskel | Herb | Nv | GG 075 |
|  | *Carthamus tinctorius* L. | Suf | Herb | FL | GG 018 |
|  | *Guizotia abyssinica*  (L.f) Casso. | Nihug | Herb | Fl | GG 012 |
|  | *Laggera tomentosa* (Sch. Bip. ex A. Rich.) | Konshkonsho | Shrub | Nv | GG 040 |
|  | *Silybum marianum* (L) Gaertn | Dander | Shrub | Nv | GG 077 |
|  | *Vernonia amygdalina* Del. | Grawa | Shrub | Nv & Hg | GG 059 |
| Balsaminaceae | *Impatiens rothii* Hook. f | Elam | Herb | Nv | GG 089 |
| Bignoniaceae | *Stereospermum kunthianum* Cham*.* | Adgizana | Shrub | Nv | GG 102 |
| Boraginaceae | *Cordia africana* Lam. | Awhi | Tree | Hg | GG 099 |
|  | *Cynoglossum lanceolatum* Forssk. | Teng-Begie | Shrub | Nv | GG 155 |
| Brassicaceae | *Brassica carinata* A.Br. | *(*Adri/senafich) | Herb | FL &HG | GG 109 |
|  | *Lepidium sativum* L. | Shnfae | Herb | FL & Hg | GG 145 |
| Buddlejaceae | *Nuxia congesta*  KBr. ex Fresen*.* | Atkaro | Tree | Nv | GG 114 |
| Cactaceae | *Opuntia ficus-indica* (L.) Miller | qulqwalbahri | Shrub | Hg&Nv | GG 037 |
| Capparidaceae | *Boscia angustifolia*  A. Rich. | Kermed | Tree | Nv | GG 046 |
|  | *Capparis tomentosa* Lam | Andel | Tree | Nv | GG 097 |
| Caricaceae | *Carica papaya* L. | Papaya | Herb | Hg | GG 127 |
| Celastraceae | *Calha edulis* (Vahl) Forssk. ex Endl. | Chat | Shrub | Nv | GG 085 |
|  | *Maytenus senegalensis* (Lam.) Exell | Argudi | Shrub | NV | GG 172 |
| Chenopodiaceae | *Beta vulgaris* L. | Keysur | Herb | Hg | GG 173 |
|  | *Chenopodium ambrosioides* L. | Etse-farus | Climber | Nv | GG 020 |
|  | *Chenopodium oplifolium* Schrader ex Koch & Ziz. | Hamli –kbo | Herb | Fl | GG 067 |
| Colchicaceae | *Gloriosa simplex* L. | Tslal-enymariam | Herb | Nv | GG 168 |
| Compositae | *Psiadia punctulata* (DC.) Vatke | Alakit | Shrub | Nv | GG 098 |
| Convolvulaceae | *Ipomoea batatas (*L.) Lam. | sekuar dinch | Herb | Hg | GG 157 |
| Crassulacea | *Kalanchoe schimperiana*  A. Rich. | Dekaeta | Herb | Nv | GG 006 |
| Cucurbitaceae | *Cucumis ficifoilus*  A. Rich. | Enkefta | Shrub | Nv | GG 084 |
|  | *Cucurbita pepo* L*.* | Duba | Herb | Hg | GG 086 |
|  | *Lagenaria siceraria* (Molina) Standl. | Amham | Herb | FL &HG | GG 112 |
|  | *Zehneria scabra* (L.f.) Sonder | Hafaflo | Shrub | Nv | GG 066 |
|  | *Zehneria anomala*  C. Jeffrey | Hareg-resa | Climber | Nv | GG 053 |
| Cuprusaceae | *Juniperus procera*  Hochst. ex Endl. | Tshdi-habesha | Tree | Nv | GG 165 |
| Cyperaceae | *Cyperus dichroostachyus*  A. Rich | Hazhaz-Anchewa | Climber | Nv | GG 047 |
| Dracaenaceae | *Sansevieria ehrenbergii* Schweinf. ex Baker | Eka | Shrub | Hg & Nv | GG 088 |
| Ebenaceae | *Euclea racemosa* Murr. ssp. *schimperi* (A.DC.) White. | Kliaw | Shrub | NV | GG 042 |
| Euphorbiaceae | *Clutia abyssinica* Jaub. & Spach | Tish bealalti | Shrub | Nv | GG 022 |
|  | *Croton macrostachyus* Del. | Tanbuk | Tree | Nv | GG 150 |
|  | *Euphorbia abyssinica* J.F.Gmel. | Kulkale | Shrub | Hg & Nv | GG 024 |
|  | *Euphorbia tirucalli* L. | Knchib | Shrub | Hg &Nv | GG 026 |
|  | *Ricinus communis* L. | Guli | Herb | Hg | GG 063 |
|  | *Tragia pungens* (Forsk.) Mull.Arg. | Am-a | Shrub | Nv | GG 104 |
| Fabaceae | *Acacia abyssinica* J.P.M | Chiea | Tree | Nv | GG 073 |
|  | *Acacia etbaica* Schweinf*.* | Seraw | Tree | Nv | GG 135 |
|  | *Acacia mellifera*  (Vahl) Benth*.* | Kerets | Tree | Nv | GG 061 |
|  | *Acacia polyacantha* Willd*.* | Gemero | Tree | NV | GG 068 |
|  | *Acacia lahai*  Steud. & Hochst. ex Benth | Lehay | Tree | Nv | GG 034 |
|  | *Albizia gummifera*  (I.F. Gmel.) C.A. Sm*.* | Sasa | Shrub | Nv | GG 131 |
|  | *Arachis hypogeal* L. | Acholoni | Herb | Fl | GG 101 |
|  | *Calpurnia aurea* (Ait) Benth | Htsawts | Shrub | Nv | GG 056 |
|  | *Cicer cuneatum*  Hochst*.* ex A. Rich. | shmbra-gwasot | Herb | Nv | GG 011 |
|  | *Cicer arietinum* L. | Shmbra | Herb | Fl | GG 152 |
|  | *Colutea abyssinica*  Kiuath & Bouche | Kokaeta | Herb | Nv | GG 048 |
|  | *Crotalaria incana* L. | hawwi-leyti | Shrub | Nv | GG 062 |
|  | *Lens culinaris* Medik. | Brsn | Herb | Fl | GG 078 |
|  | *Medicago polymorpha* L*.* | Teneg(Tsaeda | Herb | Nv | GG 003 |
|  | *Pterolobium stellatum*  (Forssk.) Brenan | Konteftefe | Shrub | Nv | GG 025 |
|  | *Senna baccarinii* (Chiov.) Lock | Hanbahanbo | Shrub | Nv | GG 060 |
|  | *Trigonella foenum-graecum* L. | Abaek | Herb | Fl | GG 094 |
|  | *Vicia faba* L. | Balenga | Herb | Fl | GG 095 |
|  | *Vigna unguiculata* (L)Walp. | Adagura | Herb | Fl | GG 108 |
| Flacourtiaceae | *Dovyalis abyssinica* (A.Rich.) Warb. | Mengolats | Shrub | Nv | GG 023 |
| Guttiferae | *Hypericum annulatum*  Moris | Hndkudkuk | Herb | Nv | GG 017 |
| Lamiaceae | *Becium grandiflorum* (Lam.) | Tebeb | Shrub | Nv | GG 142 |
|  | *Becium obovatum* (E. May. ex Benth) | Tehag | Herb | Nv | GG 153 |
|  | *Mentha polegium* L. | Setisemhal | Herb | Nv &Hg | GG 121 |
|  | *Meriandra dianthera* (Roth, ex. Roem. & Schult.) | Meseguh | Shrub | Nv | GG 004 |
|  | *Ocimum lamiifolium* Hochst. ex Benth. | Dem-kasea | Shrub | Hg | GG 071 |
|  | *Ocimum basilicum* L | Seseg | Herb | hg | GG 123 |
|  | *Otostegia integrifolia* Benth. | Chendog | Shrub | Nv | GG 082 |
|  | *Plectranthus ornatus* Codd. | Endfdf | Herb | Nv | GG 007 |
|  | *Thymus schimperi* Ronniger | Tosign | Herb | Nv | GG 151 |
| Linaceae | *Linum usitatissimum* L*.* | Entatie | Herb | Fl | GG 070 |
| Lobeliaceae | *Lobelia giberroa* Hems | Grhan | Shrub | Nv | GG 049 |
| Loganiaceae | *Buddleja polystachya* Fresen. | Metere | Tree | Nv | GG 124 |
| Malvaceae | *Hibiscus ludwigii* | Sgot | Shrub | Nv | GG 125 |
|  | *Malva verticillata* L. | Lhtit | Herb | Nv | GG 028 |
|  | *Sida schimperiana* Hochst. ex A.Rich | Tfrerya | Shrub | Nv | GG 158 |
| Meliaceae | *Ekebergia capensis*  Spamn. | Kot | Tree | Nv | GG 031 |
|  | *Melia azedarach* L. | Niem | Tree | Hg | GG 122 |
| Melianthaceae | *Bersama abyssinica*  Fresen | Asha-om | Shrub | Nv | GG 111 |
| Mimosoideae | *Acacia albida* Del. | Momona | Tree | Nv | GG 126 |
| Moraceae | *Ficus glumosa* Del. | Chekente | Tree | Nv | GG 090 |
|  | *Ficus palmata* Forssk. | Beles/demay | Tree | Nv | GG 005 |
|  | *Ficus sur* Forssk*.* | Kodo | Tree | Nv | GG 041 |
|  | *Ficus vasta* Forssk. | Daero | Tree | Nv | GG 091 |
| Moringaceae | *Moringa oleifera* Lam*.* | Shefraw | Shrub | Hg | GG 141 |
| Musaceae | *Musa paradisiaca* L. | Muz | Herb | Hg | GG 136 |
| Myricaceae | *Myrica salicifolia* A.Rich. | Nebi | Tree | Nv | GG 116 |
| Myrsinacea | *Maesa lanceolata* Forssk. | Sewerya | Tree | Nv | GG 138 |
| Myrtaceae | *Eucalyptus globulus* Labill. | Tsaeda-kelamitos | Tree | Hg | GG 144 |
|  | *Syzygium guineense* (Willld.) Dc | Liham | Tree | Nv | GG 033 |
| Oleaceae | *Jasminum abyssinicum* Hochst. ex DC. | Habitselim | Shrub | Nv | GG 051 |
|  | *Olea europaea* L. ssp. *caspidata* (Wall. ex G.Don.) | Awlie | Tree | Nv | GG 105 |
| Oxalidaceae | *Oxalis anthelmintica*  A. Rich. | Habichego | Herb | Nv | GG 015 |
| Papaveraceae | *Argemone mexicana* L. | medafe-t'ilian | Herb | Nv | GG 161 |
| Phytolaccaceae | *Phytolacca dodecandra* L’Herit. | Shbti | Shrub | Nv | GG 117 |
| Plumbaginaceae | *Plumbago zylanica* L. | Aftuh | Shrub | Nv | GG 107 |
| Poaceae | *Eleusine floccifolia*  (Forssk.) Spreng. | Rghe | Herb | Nv | GG 137 |
|  | *Hordeum vulgare* L | Sgem/bukuli | Herb | Fl | GG 130 |
|  | *Sorghum bicolor* (L.) Monch | Mashla | Herb | Fl | GG 019 |
| Podocarpaceae | *Podocorpus falcatus* (Thunb.Mrib) | Zgba | Tree | Nv | GG166 |
| Po1ygonaceae | *Rumex nepalensis* Spreng*.* | Shenbwaeta | Shrub | Nv | GG 146 |
|  | *Rumex nervosus* Vahl | Hihot | Shrub | Nv | GG 054 |
|  | *Oxygonum sinuatum* Dammer | Chew-murakut | Herb | Nv | GG 093 |
|  | *Rumex abyssinicus* Jacq. | Mekmoko | Herb | Nv | GG 035 |
| Ranunculaceae | *Clematis simensis* Fresen*.* | Hareg | Climber | Nv | GG 053 |
|  | *Dichrostachys cinerea* | Gonek | Shrub | Nv | GG 052 |
|  | *Nigella sativa* L. | Awesda/tkur azmud | Herb | Hg | GG 106 |
| Rhamnacea | *Rhamnus prinoides* L’Herit | Gesho | Shrub | Hg | GG 043 |
|  | *Ziziphus spina-christi* (L.) Desf. | Gaba | Tree | Nv | GG 072 |
| Rosaceae | *Hagenia abyssinica* (Bruce) J.F. Gmel. | Habi | Tree | Nv | GG 065 |
|  | *Prunus persica* (L.) Batsch | Kuk | Shrub | Hg | GG 032 |
|  | *Rosax richardii* Rehd | Tsgereda | Shrub | Hg | GG 162 |
| Rubiaceae | *Coffea arabica* L. | Buna | Shrub | Hg | GG 074 |
| Rutaceae | *Citrus limon* (L.) Burm.f. | Lemin | Shrub | Hg | GG 171 |
|  | *Citrus medical* L. | Trngi | Shrub | Hg | GG 154 |
|  | *Citrus sinensis*  (L.) Osb. | Brtukan | Shrub | Hg | GG 081 |
|  | *Ruta Chalepensis* L. | Chena-Adam | Herb | Hg | GG 092 |
| Sapindacea | *Dodonea angustifolia*e L.f | Tahses | Shrub | Nv | GG 140 |
| Sapotaceae | *Mimusops kummel* A.DC. | Kumel | Tree | Nv | GG 030 |
|  | *Sideroxylon oxyacanthum* Baill | Seroro | Tree | Nv | GG 118 |
| Scrophulariaceae | *Verbascum sinaiticum* Benth. | Trnaka | Tree | Nv | GG 159 |
| Simaroubaceae | *Brucea antidysenterica*  J.F. Mill. | (Maleta | Shrub | Nv | GG 021 |
| Solanaceae | *Capsicum annuum* L. | Berber | Herb | Hg | GG 069 |
|  | *Capsicum frutescens* L | Mitmita | Herb | Hg | GG 119 |
|  | *Datura stramonium* L. | Astenagr | Herb | Nv | GG 096 |
|  | *Discopodium penninervium* Hochst. | Gaeta | Shrub | Nv | GG 079 |
|  | *Lycopersicon esculentum* Mill. | Kumedre | Herb | Hg | GG 027 |
|  | *Nicotiana tabacum* L. | Tnbako | Herb | Nv | GG 148 |
|  | *Solanum incanum* L | Engule | Shrub | Nv | GG 002 |
|  | *Withania somnifera* L. | Agol | Shrub | Nv | GG 016 |
| Sterculiaceae | *Dombeya torrida*  (J. F. Gmel.) P. Bamps | Tsnkuya | Shrub | Nv | GG 164 |
| Verbenaceae | *Clerodendron myricoides*  (Hochst.) Vatke | Surbetry | Shrub | Nv | GG 014 |
|  | *Verbena officinalis* L. | Atuch | Shrub | Nv | GG 110 |
| Vitaceae | *Cissus petiolata*  Hook.f. | Alke | Climber | Nv | GG 115 |
|  | *Vitis vinifera* L. | Weyni | Climber | Hg | GG 167 |
| Zingiberaceae | *Aframomum corrorima*  *(*Braun) Jansen | Korerima | Herb | Hg | GG 039 |
|  | *Curcuma domestica* Valeton | Erdi | Herb | Hg | GG 083 |
|  | *Zingiber ofjicinale* Rosc. | Zngbl | Herb | Hg | GG 163 |
| Zygophyllaceae | *Tribulus terrestris* L. | Kakito | Herb | Nv | GG 050 |
