# Supplementary material for: Ethnobotanical study of medicinal plants in Ganta Afeshum District, Eastern Zone of Tigray, Northern Ethiopia
Source: J Ethnobiol Ethnomed. 2018 Nov 3;14:64. doi: 10.1186/s13002-018-0266-z (PMC6215673; doi:10.1186/s13002-018-0266-z)
Supplement: Supplementary file 3 — Table S3. List of medicinal plants used for treating human ailments. (DOC 402 kb) [file 13002_2018_266_MOESM3_ESM.doc]

**Table S3. List of medicinal plants used** for treating human ailments

| **Family name** | **Species name** | **Local name** | **Habit** | **Method of preparation and application; condition of preparation and part used** | **Ailments treated** |
| --- | --- | --- | --- | --- | --- |
| Acanthaceae | *Achyranthes aspera* | Mchelo | Herb | Fresh leaf and stem is crushed and smear on head. | Tonsillitis |
|  |  |  |  | Fresh root is crushed, mixed with black faeces, filtered and the dropped in eye. | Eye disease |
|  | *Hypoestes forskaolii* | Grbya | Herb | Dry/fresh, root of the medicinal plant is tied on the damaged part of the body. | Dislocated bone |
|  | *Justicia schimperiana* | Shimeza | Shrub | Fresh leaf is crushed, mixed with water, filter and drink. | Jaundice/ Efshwa |
| Alliaceae | *Allium cepa* | Keyh-shgurti | Herb | Fresh bulb is crushed, mixed with honey; allow staying for seven days and then eating at morning before eating food until recovery. | Asthma |
|  | *Allium sativum* | Tsaeda-shgurti | Herb | Unprocessed fresh bulb eat directly | Impotence in male, Cough and Pain after delivery |
|  |  |  |  | Fresh bulb is crushed, mixed with honey and eat at morning for seven days. | Asthma |
|  |  |  |  | Fresh bulb is crushed, mixed with water, filter and sniff through nose | Head ach |
|  |  |  |  | Fresh leaf is crushed, mixed with butter and smear. | Paralysis |
|  |  |  |  | Chewing fresh bulb is a treatment for teeth ach. | Teeth ach |
| Aloaceae | *Aloe camperi* | Sandaere | Herb | Fresh latex is directly dropped in eye. | Eye disease |
|  | *Aloe megalacantha* | Ere | Herb | Fresh/dry root is tied on hand/leg/body. | dislocated bone |
|  |  |  |  | Fresh latex is squeezed from young upper part and drink. | Malaria |
|  |  |  |  | Fresh fruit is crushed, mixed with water and smeared in the anus. | Hemorrhoid |
| Amaranthaceae | *Aervaja vanica* | Lge-Dmu | Shrub | Fresh latex is directly smeared on the infected part of the skin. | Coetaneous lishmaniasis |
|  | *Alternanthera nodiflora* | Kodo-Gih/Tetem –gih | Shrub | Fresh whole plant is crushed and rubbed at the site of bite. | Snake bite |
|  | *Amaranthus caudatus* | Mendef-Adgi/ Hamli-adgi) | Shrub | Chewing fresh root can treat for teeth ach | Teeth ach |
| Anacardiaceae | *Rhus glutinosa* | Tetael | Tree | Fresh leaf is boiled in water and drinks it at morning before eating food. | Ascariasis |
|  | *Schinus molle* | Tkur-berbere | Tree | Fresh leaf is crushed, mixed with water, filtered and drink at the time of pain. | Jaundice, Tape worm, |
|  |  |  |  | Fresh leaf is crushed, mixed with water, filtered and sniff through nose. | Head ach, Evil eye/ |
|  |  |  |  | Fresh leaf is crushed, mixed with water, filtered and sniff through nose. | Diarrhea $ vomiting |
| Apiaceae | *Conium maculatum* | Tsakda | Herb | Chewing unprocessed dry seed is a treatment for abdominal pain | Abdominal pain |
|  | *Cuminum cyminum* | Kemun | Herb | Chewing dry seed is treatment for abdominal pain | Abdominal pain |
|  | *Dacus carota* | Carot | Herb | Fresh leaf is boiled in water and drink as tea. | *Head ach* |
|  | *Foeniculum vulgare* | Shlan | Herb | Fresh leaf is crushed, mixed with water, filter and drink. | Urine retention/ atsre shnti |
|  | *Heteromorpha arborescens* | Seseg –zbe | Herb | Fresh bulb is crushed, mixed with honey and swallow. | Head ach |
|  | *Trachyspermum ammi* | Azmud | Herb | Fresh and dry leaf is boiled in water and drink | Asthma |
| Apocynaceae | *Acokanthera schimperi* | Mebte | Tree | Fresh/dry bark is powdered, heated on oven, mixed with butter smeared on body skin. | Skin rash/hafew |
|  |  |  |  | Fresh $ dry root is burnt $ fumigate by its steam. | Evil eye |
|  |  |  |  | Dry root is powdered, mixed with goat butter and smear on affected body. | Wound |
|  |  |  |  | Fresh leaf is crushed, mixed with honey and smear at anus. | Hemorrhoid |
|  | *Calotropis procera* | Gindae | Herb | Fresh latex is smeared on affected body the body. | Wound |
|  | *Carissa spinarum* | Agam | Tree | Dry root and bark is burnt and fumigate by its smoke. | Evil eye |
|  |  |  |  | Dry and fresh root is burnt and fumigate by its smoke. | Evil spirit |
|  |  |  |  | Fresh leaf is boiled in water and drunk. | Diabetes |
| Arecaceae | *Borassus aethiopum* | Sye | Tree | Dry fruit is powdered, mixed with sesame oil and then dropped in to ear. | Ear infection. |
| Asclepiadaceae | *Dregea abyssinica* | Shankuk | Shrub | Fresh leaf is crushed, mixed with water and smear on the affected body. | Mumps |
|  | *Periploca linearifolia* | Moder | Tree | Dry root is burnt in fire and fumigate by its smoke. | Evil spirit |
|  | *Gomphocarpus fruticosus* | Demayto | Herb | Fresh root is chewed and suck the liquid. | Abdominal pain |
|  |  |  |  | Paint and rub the infected body by latex of the plant. | Cutaneous Leshimaniasis |
| Asparagaceae | *Agave americana* | Eka-trmo/eka tlyan | Shrub | Fresh bark is crushed smeared on the infected body. | Cutaenous lishmaniasis & ring worm |
|  | *Asparagus africanus* | Kesta-Ansti | Shrub | Dry and fresh root is burnt in fire and fumigate by its smoke. | Evil eye |
|  |  |  |  | Fresh leaf and root is crushed, mixed with water and smear on to anus. | Hemorrhoid |
| [Asteraceae](https://en.wikipedia.org/wiki/Asteraceae) | *Artemisia abyssinica* | ChenaBarya/wedwado | Herb | Fresh and dry stem is brunt and fumigate by its smoke. | Evil eye. |
|  |  |  |  | Smelling fresh leaf is a treatment for cough. | Cough |
|  | *Vernonia amygdalina* | Grawa | Shrub | Fresh leaf is crushed mixed with water, filter and drink | Fibril illness |
|  |  |  |  | Dry leave is powdered, mixed with honey(brzi) and drink | Impotence in male |
|  |  |  |  | Fresh leaf is crushed, filtered and sniffed through nose. | Evil eye |
|  | *Guizotia abyssinica* | Nihug | Herb | Dry seed is powdered, boiled in honey and drink. | Heart disease |
|  | *Bidens macroptera* | Gelgele-meskel | Herb | Dry flower crushed and smell | Head ach |
|  | *Carthamus tinctorius* | Suf | Herb | Dry seed is crushed and squeezed from it and then drink. | Asthma |
|  | *Laggera tomentosa* | Konshkonsho | Shrub | Fresh leaf is boiled in water and fumigate by its steam | fibril illness |
|  |  |  |  | Dry root is powdered and sniff through nose. | Bleeding /nesri |
|  | *Silybum marianum* | Dander | Shrub | Fresh root is crushed, mixed with honey and swallow. | Impotence in male |
| Balsaminaceae | *Impatiens rothii* | Elam | Herb | Fresh bulb crushed and smeared on hands | Arthritis |
| Bignoniaceae | *Stereospermum kunthianum* | Adgizana | Shrub | Dry bark is crushed and powdered, mix with honey then smear on affected dermis. | Wound |
| Boraginaceae | *Cordia africana* | Awhi | Tree | Fresh leaf is crushed, mixed with tea/coffee and drink. | Fibril illness |
|  |  |  |  | Fresh bark is crushed, mixed with butter and eat. | Abortion |
|  |  |  |  | Fresh leaf and root is crushed and smeared in anus. | Hemorrhoid |
|  |  |  |  | Fresh fruit is swallowed to release amoeba with faeces. | Amoeba |
| Brassicaceae | *Lepidium sativum* | Shnfae | Herb | Dry seed is powdered, mixed with ergo and the drink. | Bloody diarrhea |
|  |  |  |  | Dry seed is powdered and put on the wound. | Wound |
|  |  |  |  | Dry seed is powdered mixed with butter and smear on affected body. | Paralysis/ gusay |
|  |  |  |  | Dry seed is powdered, mixed with fat of snake and then smear on the skin. | Skin problem; locally called Lmtsi |
|  | *Brassica carinata* | *(*Adri/senafich) | Herb | Dry seed is powdered; mix with water and then drink. | Constipation $ blood pressure |
|  |  |  |  | Fresh seed is crushed; mix with fat of snake and then smear on the skin. | Skin problem; locally called Lmtsi |
|  |  |  |  | Dry seed is crushed, mix with honey and drunk | Cough |
| Buddlejaceae | *Nuxia congesta* | Atkaro | Tree | Fresh leaf is crushed and mixed with sesame oil and dropped in to ear. | Ear infection |
| Cactaceae | *Opuntia ficus-indica* | qulqwalbahri | Shrub | Dry and fresh root is tied on hand/leg | Dislocated bone |
| Capparidaceae | *Boscia angustifoliae* | Kermed | Tree | Dry and fresh bark is cut and ties on body. | Evil spirit |
|  | *Capparis tomentosa* | *Andel* | Tree | Dry root is burnt and fumigate by its steam. | Evil spirit |
|  |  |  |  | Fresh leaf is crushed and smeared on breast. | Breast diseases |
| Caricaceae | *Carica papaya* | Papaya | Herb | Unprocessed and fresh fruit is eaten. | Constipation |
|  |  |  |  | Fresh leaf is boiled in water and drink. | Gastritis |
| Celastraceae | *Calha edulis* | Chat | Shrub | Fresh leaf is crushed and smeared on the body. | Irritation |
|  | *Maytenus senegalensis* | Argudi | Shrub | Fresh leaf is crushed, mixed with butter and smeared in and around anus. | Hemorrhoids |
|  |  |  |  | Un processed fresh leaf is chewed. | Scorpion bite |
| Chenopodiaceae | *Beta vulgaris* | Keysur | Herb | Fresh bulb is crushed, boiled in water and drink at sleep. | Cough |
|  | *Chenopodium ambrosioides* | Etse-farus | Climber | Fresh/dry root is crushed, mixed with honey and swallow | Snake bite |
| *Compositae* | *Psiadia punctulata* | Alakit | Shrub | Fresh leaf is crushed, mixed with *Allium sativum* and smeared on infected dermis. | Herpes/ Almaz balechra/ |
|  |  |  |  | Fresh leaf is crushed and smeared on the affected skin. | Fire burn-wound |
|  |  |  |  | Fresh and dry leaf is burnt and fumigate by its smoke. | fibril illness |
| Convolvulaceae | *Ipomoea batatas* | sekuar dinch | Herb | A fresh bulb is boiled in water and then eats the bulb. | Heavy tiredness |
| Crassulacea | *Kalanchoe schimperiana* | Dekaeta | Herb | Fresh leaf is heated in fire and press on the breast. | breast disease |
| Cucurbitaceae | *Cucumis ficifoilus* | Enkefta | Shrub | Fresh leaf is crushed, mixed with tella/milk and drink. | Jaundice/ Efshwa |
|  | *Zehneria anomala* | Hareg-resa | Climber | Fresh leaf is boiled in water and drink. | Head ach |
|  | *Zehneria scabra* | Hafaflo | Shrub | Fresh leaf is boiled in water and fumigate by its smoke. | fibril illness$ cough |
|  |  |  |  | Chewing fresh leaf is a treatment for Bud smelling of mouth | Bud smelling of mouth |
|  | *Lagenaria siceraria* | Amham | Herb | Fresh leaf is crushed, mixed with water and honey and drink. | Fear $dislike of sex in females |
|  |  |  |  | Fresh leaf is crushed, filtered by cotton and dropped in to ear. | Ear infection |
|  | *Cucurbita pepo* | Duba | Herb | Dry seed is roasted and eat as kolo. | Tape worm |
|  |  |  |  | Fresh fruit cooked and eat as food. | Constipation |
| Cuprusaceae | *Juniperus procera* | Tshdi-habesha | Tree | Dry seed is powdered, mixed with water and honey and drink. | Fear $dislike of sex in females |
| Cyperaceae | *Cyperus dichroostachyus* | Hazhaz-Anchewa | Climber | Fresh leaf is crushed and smear on infected body | Cutaneous Leshimaniasis |
| Dracaenaceae | *Sansevieria ehrenbergii* | Eka | Shrub | Fresh bark is heated in fire and press on the affected body. | Nasal disease |
| Ebenaceae | *Euclea racemosa* | Kliaw | Shrub | Dry root is powdered, mixed in water and swallow. | *Tonsillitis* |
|  |  |  |  | Chewing fresh and dry root is a treatment for teeth ach. | Teeth ach |
|  |  |  |  | Fresh and dry root is crushed, mixed with butter and smear on face. | black spot on face/ Madyat |
| Euphorbiaceae | *Tragia pungens* | Am-a | Shrub | The infected skin is rubbed by fresh leaf | *Ring worm* |
|  | *Croton macrostachyus* | Tanbuk | Tree | Fresh leaf is crushed mixed with tella/milk and drink. | Jaundice |
|  | *Euphorbia tirucalli* | Knchib | Shrub | Fresh latex is smeared on the affected body. | Wound |
|  | *Clutia abyssinica* | Tish bealalti | Shrub | Dry and fresh of the whole plant is burnt and fumigate by its smoke to vagina during pregnancy and after birth. | Vaginal infection |
| Fabaceae | *Acacia albida* | Momona | Tree | Fresh and dry bark is crushed, mixed with bile of sheep and smear on affected area of the skin. | Wound |
|  | *Acacia etbaica* | Seraw | Tree | Dry leaf is powdered, mixed with butter and smear on head. | Head wound |
|  |  |  |  | When dry/fresh stem is burnt latex produced and the smear by the latex on affected area of the skin. | Fungal infection/ tewsas |
|  | *Acacia mellifera* | kerets | Tree | Dry root is powdered, mixed with honey and eat. | Lepros*y* |
|  |  |  |  | Fresh leaf is crushed, mixed with honey and swallow. | Rhfactor/ Mengegna |
|  | *Acacia polyacantha* | Gemero | Tree | Dry and fresh root crushed, burnt and fumigate by its smoke. | Evil spirit |
|  | *Acacia* *lahai* | Lehay | Tree | Fresh fruit is crushed and smeared on face. | Granule/ Begur/ Fetsega |
|  | *Albizia gummifera* | Sasa | Shrub | Fresh leaf is boiled in water and fumigate by its steam. | Fibril illness |
|  | *Arachis hypogea* | Acholoni | Herb | Dry seed is powdered, mix with tea and then drink | Heart disease |
|  | *Calpurnia aurea* | Htsawts | Shrub | Fresh leaf is crushed with water, filter by using cotton and dropped in sick eye. | Eye disease |
|  | *Cicer cuneatum* | shmbra-gwasot | Herb | Chewing fresh root is a treatment for abdominal pain | Abdominal pain |
|  | *Cicer arietinum* | Shmbra | Herb | Dry seed is boiled in water and the drink at sleep. | Impotence in male |
|  | *Colutea abyssinica* | Kokaeta | Herb | Unprocessed fresh leaf directly is eaten. | Tuberculosis |
|  | *Lens culinaris* | Brsn | Herb | Dry seed is boiled in water and drink the liquid portion.. | Impotence in male |
|  | *Medicago polymorpha* | Teneg (Tsaeda) | Herb | Fresh leaf is crushed, mixed with tea/coffee and drink. | Fibril illness |
|  | *Pterolobium stellatum* | Konteftefe | Shrub | Dry and fresh root is burnt and fumigate by its smoke. | Evil eye |
|  | *Senna baccarinii* | Hanbahanbo | Shrub | Dry root is powdered, mixed with butter and smear on the body. | Skin rash/ hafew |
|  | *Vicia faba* | Balenga | Herb | Dry seed is crushed and smeared on the swelled skin. | Wound /Megli-anchwa |
|  | *Trigonella foenumgraecum* | Abaeke | Herb | Dry seed is powdered, mix with honey and eat. | Asthma and Cough |
|  |  |  |  | Dry seed is powdered, mixed with butter and smear on the affected dermis. | Wound, locally called Megli anchwa |
| Flacourtiaceae | *Dovyalis abyssinica* | Mengolats | Shrub | Fresh leaf is crushed, mixed with the oil of *Guizotia abyssinica* and eat | Tape worm |
| Guttiferae | *Hypericum annulatum* | Hndkudkuk | Herb | Fresh leaf is crushed, mixed with water and drink. | Gonorrhea |
| Lamiaceae | *Becium grandiflorum* | Tebeb | Shrub | Dry stem is burnt and fumigate by its smoke. | Evil eye and evil spirit |
|  | *Mentha polegium* | Setisemhal | Herb | Chewing fresh leaf and stem is treatment for teeth ach. | Teeth ach |
|  | *Meriandra dianthera* | Meseguh | Shrub | Fresh leaf is crushed, filter and drink. | Abdominal pain |
|  | *Ocimum lamiifolium* | Dem-kasea | Shrub | Fresh leaf is crushed, filtered, drink alone/with tea/with coffee. | Fibril illness |
|  | *Thymus schimperi* | Tosign | Herb | Fresh and dry leaf is mixed with honey and boiled and then drink. | Abdominal pain |
|  | *Plectranthus ornatus* | Endfdf | Herb | Fresh and dry root is tied on hand/ leg/body with cotton that never soaked in water before. | Dislocated bone |
|  |  |  |  | Fresh root is crushed, mixed with water and smear on head. | Tonsillitis |
|  | *Otostegia integrifolia* | Chendog | Shrub | Fresh leaf is crushed, mixed with water and drink. | Amoeba |
|  |  |  |  | Chewing fresh leaf is a treatment for throat infection. | Throat infection |
| Linaceae | *Linum usitatissimum* | Entatie | Herb | Dry seed is powdered, mixed with water, boil and drink. | Constipation |
|  |  |  |  | Dry seed is soaked in water for three days and then swallow | Amoeba |
| Lobeliaceae | *Lobelia giberroa* | Grhan | Shrub | Dry leaf is powdered and sniffed through nose. | Evil eye |
| Loganiaceae | *Buddleja polystachya* | Metere | tree | Fresh leaf is crushed, filter and drink. | Tonsillitis |
| Malvaceae | *Hibiscus ludwigii* | Sgot | Shrub | Fresh leaf is crushed, mixed with honey and smear. | Body swelling locally called Megerem. |
|  | *Sida schimperiana* | Tfrerya | Shrub | Chewing fresh root at time of pain is a treatment for | Abdominal pain |
|  | *Malva verticillata* | Lhtit | Herb | Fresh leaf is crushed, filtered and dropped in to ear. | Ear infection |
|  |  |  |  | Fresh leaf is crushed and sniffed through nose. | Head ach |
| Meliaceae | *Ekebergia capensis* | Kot | Tree | Fresh bark is boiled in water and wash at morning. | Skin disease |
|  | *Melia azedarach* | Niem | Tree | Fresh leaf is crushed, mixed with water and drink. | Malaria |
| Moraceae | *Ficus sur* | Kodo | Tree | Fresh bark is boiled in water and wash. After washing not expose on the sun. | Skin disease |
|  | *Ficus vasta* | Daero | Tree | Fresh is crushed, filtered and dropped in eye. | Eye disease |
|  |  |  |  | Fresh bark is boiled in water and wash at morning. After washing not allowed exposing on sun. | Skin disease |
|  | *Ficus palmata* | Beles/demay | Tree | Fresh and dry root is heated in fire and pressed on affected skin. | Body swelling |
|  |  |  |  | Dry and fresh stem is tied around the body. | Abortion |
|  |  |  |  | The latex of the plant is directly dropped in to the ear. | Ear infection |
|  |  |  |  | The infected dermis is rubbed by fresh leaf. | Ring worm |
| Moringaceae | *Moringa oleifera* | Shefraw | Shrub | Fresh root is crushed, mixed with the oil of *Carthamus tinctorius* and the drink. | Asthma |
|  |  |  |  | Fresh leave is crushed, mixed with water and drink. | Blood pressure |
| Musaceae | *Musa paradisiaca* | Muz | Herb | Fresh bark is crushed, mixed with butter and smear at anus. | Hemorrhoid |
| Myricaceae | *Myrica salicifolia* | Nebi | Tree | Dry bark is powdered and sniff through nose. | Tumor/ Menkersa and head ach. |
| Myrsinacea | *Maesa lanceolata* | Sewerya | Tree | Dry fruit is powdered, heat on oven, mix with butter and smear on the body. | Skin rash |
| Myrtaceae | *Eucalyptus globulus* | Tsaeda-kelamitos | Tree | Fresh leaf is boiled in water and fumigate by its steam. | Feberileillne and Cough |
|  | *Syzygium guineense* | Liham | Tree | Fresh bark is boiled in water and drink at morning before eating food. | Diarrhea |
| Oleaceae | *Jasminum abyssinicum* | Habitselim | Shrub | Fresh leaf is grind, filtered by using cotton and dropped in eye. | Eye disease |
|  | *Olea europaea* | Awlie | Tree | Fresh leaf is boiled in water and drink before eating food. | Abdominal pain $ Head ach |
|  |  |  |  | Fresh leaf is crushed, mixed with butter and smeared in anus. | Hemorrhoids |
| Oxalidaceae | *Oxalis anthelmintica* | Habichego | Herb | Fresh bulb of *Oxalis anthelmintica* is crushed mixed with *Barleria grandicalyx and* swallow. | Tapeworm |
| Papaveraceae | *Argemone mexicana* | medafe-t'ilian | Herb | Cut the apex part of the plant and smear by latex to the wound. | Wound |
| Phytolaccaceae | *Phytolacca dodecandra* | Shbti | Shrub | Fresh leaf is crushed, mixed with honey and eat. Drinking tella and eating meat is not allowed. | Body swelling locally called megrem |
|  |  |  |  | Fresh root is crushed, mixed with tella and drink. | Rabies |
| Plumbaginaceae | *Plumbago zylanica* | Aftuh | Shrub | Dry and fresh root is burnt and fumigate by its smoke. | The diseases is locally called Sray/dgam |
|  |  |  |  | Fresh leaf is crushed, mix with water and wash every morning for seven days. | Evil eye |
| Poaceae | *Eleusine floccifolia* | Rghe | Herb | Dry and fresh root is tied by new cotton. | Dislocated bone |
|  | *Hordeum vulgare* | Sgem/bukuli | Herb | Dry seed is roasted and eat as food. | Gastritis |
| Podocarpaceae | *Podocorpus falcatus* | Zgba | Tree | Fresh root is crushed, mixed with butter and smear. | black spot on face/ Madyat |
| Polygonaceae | *Oxygonum sinuatum* | Chew-murakut | Herb | Fresh bulb is mixed with kolo and eat | Tape worm |
|  | *Rumex nepalensis* | Shenbwaeta | Shrub | Fresh leaf is crushed, mixed with water and drink. | Tonsillitis |
|  |  |  |  | Fresh leaf is heated in fire and the rub by the heated leaf on infected part of leaf. | Ring worm |
|  | *Rumex abyssinicus* | Mekmoko | Herb | Fresh $ dry bulb is crushed, mixed with oil of *Guizotia abyssinica* and drink*.* | Prolonged delivery |
|  | *Rumex nervosus* | Hihot | Shrub | Dry leaf is powdered and tied on the body. | Evil spirit |
|  |  |  |  | Fresh root is crushed, add to teji and drink | Impotence in male |
|  |  |  |  | Fresh /dry stem is crushed, mixed with water and wash at morning for seven days. | Sray/dgam |
| Ranunculaceae | *Dichrostachys cinerea* | Gonek | Shrub | Fresh bark is tied on damaged part of the body with cotton that never washed before. | Dislocated bone |
|  | *Clematis simensis* | Hareg | climber | Fresh bark is crushed, mixed with water and smear on affected body part | Cutaneous Leshimaniasis |
|  | *Nigella sativa* | Awesda/tkur azmud | Herb | Dry seed is powdered, mixed with honey and swallowed. | Abdominal pain $ cough |
| Rhamnaceae | *Ziziphus spina-christi* | Gaba | Tree | Fresh leaf is crushed, mixed with water and wash by removing the hairs before. | Dandruff/ Forefor |
|  | *Rhamnus prinoides* | Gesho | Shrub | Chewing fresh, young part of the medicinal plant is treatment for tonsillitis | Tonsillitis |
| Rosaceae | *Rosax richardii* | Tsgereda | Shrub | Fresh flower is boiled in water, dropped in ear | Ear infection. |
|  | *Prunus persica* | Kuk | Shrub | Fresh fruit is heated and press by heated fruit on head. | Head ach |
|  | *Hagenia abyssinica* | Habi | Tree | Fresh leaf is crushed, filtered and drink. | Tapeworm |
| Rubiaceae | *Coffea arabica* | Buna | Shrub | Dry seed is crushed and smeared on the wound. | Wound |
|  |  |  |  | Fresh leaf is boiled in water and drink. | Head ach |
| Rutaceae | *Citrus limon* | Lemin | Shrub | Fresh fruit is squeezed and rubbed on affected skin. | Skin problem |
|  |  |  |  | Chewing dry root is a treatment for the disease locally called Sray /dgam | Sray /dgam |
|  |  |  |  | Fresh fruit boiled and cooked and then eat before eating food. | Pain after delivery/ hmam dehar |
|  |  |  |  | Fresh and dry bark is crushed, mixed with butter and eat. | Rabies |
|  | *Citrus medical* | Trngi | Shrub | Unprocessed fresh fruit eat directly. | Constipation |
|  | *Citrus sinensis* | Brtukan | Shrub | Fresh leaf is boiled in water and drink as tea. | Head ach |
|  | *Ruta chalepensis* | Chena-Adam | Herb | Fresh leaf is directly added in tea, coffee, milk and drink. | Cough |
| Sapindacea | *Dodonea angustifolia* | Tahses | Shrub | Fresh leaf is crushed and the smear on the affected skin. | Herpes/ Almaze balchera |
|  |  |  |  | Fresh bark is powdered, mixed with water and the drink. | Ascariasis |
|  |  |  |  | Fresh fruit is crushed mixed with honey and swallow. | Malaria |
| Sapotaceae | *Mimusops kummel* | Kumel | Tree | Fresh fruit is crushed, mixed with new milk and drink. | Measles |
|  | *Sideroxylon oxyacanthum* | Seroro | Tree | Dry fruit is powdered, heat on oven, mixed with butter and finally smear. | Skin rash |
| Scrophulariaceae | *Verbascum sinaiticum* | Trnaka | Tree | Fresh root is crushed, mixed with water, filtered and drink. | Retained placenta |
|  |  |  |  | Fresh root is crushed, mixed with water, filtered and drink. | Jaundice/ Efshwa |
| Simaroubaceae | *Brucea antidysenterica* | Maleta | Shrub | Fresh fruit is crushed, mixed with honey and smear on the affected body. | Leprosy |
| Solanaceae | *Datura stramonium* | Astenagr | Herb | Fresh leaf is crushed and smeared on skin. | Wound |
|  |  |  |  | Dry fruit is roasted on oven with butter and fumigate by its smoke. | Teeth ach |
|  | *Nicandra physalodes* | Hamli –kbo | Herb | Dry leaf is powdered; mixed with water, smear on the damaged skin. | Fire Burn Wound |
|  | *Solanum incanum* | Engule | Shrub | Dry fruit is crushed, mixed with water and smeared on the body. | Itching |
|  |  |  |  | Dry root is crushed, mixed with honey and eat. | Leprosy |
|  |  |  |  | Fresh root is chewed and liquid is sucked. | Abdominal pain |
|  |  |  |  | Dry and fresh root powdered, burnt in fire and fumigate by its smoke. | Sray/dgam |
|  |  |  |  | Fresh root is crushed, mixed with water, filter and drink | Male impotence |
|  | *Nicotiana tabacum* | Tnbako | Herb | Dry leaf is powdered; mixed with water, smear on the damaged skin. | Fire Burn Wound |
|  | *Withania somnifera* | Agol | Shrub | Fresh leaf is boiled in water and fumigate by its steam | fibril illness |
|  |  |  |  | Fresh and dry leaf and root is burnt and fumigate by its smoke. | Evil spirit |
|  |  |  |  | Fresh leaf is crushed, mix with butter then smeared on skin | Paralysis locally called gusay |
|  | *Lycopersicon esculentum* | Kumedre | Herb | Fresh leaf is crushed and smeared on head. | Tonsillitis |
|  |  |  |  | Fresh root crushed, soak in water and wash the body at morning. | Evil spirit |
|  |  |  |  | Fresh leaf is boiled in water and then drink | Urine retention |
| Sterculiaceae | *Dombeya torrida* | Tsnkuya | Shrub | Fresh bark is crushed and smear on affected body. | Fire Burn Wound |
| Verbenaceae | *Verbena officinalis* | Atuch | Shrub | Dry root is crushed and sniffed through nose. | Sray/dgam |
|  | *Clerodendron myricoides* | Surbetry | Shrub | Fresh leaf and stem is burnt in fire and fumigate by its smoke. | Feberileillne |
| Vitaceae | *Cissus petiolata* | Alke | Climber | Fresh and dry, leaf and root is crushed and eaten. | Cough |
|  |  |  |  | Fresh whole plant is crushed and soaked in cattle urine and wash for seven days. | Body swelling locally called Mendaeti |
|  | *Vitis vinifera* | Weyni | Climber | Fresh fruit is squeezing and drink. | Blood pressure, eye $heart diseases |
|  |  |  |  | Fresh fruit is squeezing, mixed with egg, boil and drink. | Cough |
| Zingiberaceae | *Zingiber ofjicinale* | Zngbl | Herb | Chewing fresh bulb is a treatment for teeth ach. | Teeth ach |
|  | *Curcuma domestica* | Erdi | Herb | Unprocessed dry seed is chewed. | Abdominal pain |
|  | *Aframomum corrorima* | Korerima | Herb | Dry bulb is powdered, mixed with milk and drink. | Urine retention/ atsre-shnti |
