# Supplementary material for: Ethnobotanical study of medicinal plants in Ganta Afeshum District, Eastern Zone of Tigray, Northern Ethiopia
Source: J Ethnobiol Ethnomed. 2018 Nov 3;14:64. doi: 10.1186/s13002-018-0266-z (PMC6215673; doi:10.1186/s13002-018-0266-z)
Supplement: Supplementary file 4 — Table S4. List of medicinal plants used for treating livestock ailments. (DOC 63 kb) [file 13002_2018_266_MOESM4_ESM.doc]

Table S4. List of medicinal plants used for treating livestock ailments

| **Family name** | **Scientific name** | **Local name** | **Habit** | **Method of preparation, application and part used** | **Ailments treated** |
| --- | --- | --- | --- | --- | --- |
| Apiaceae | *Heteromorpha arborescens* | Seseg–zbe | Herb | Fresh leaf is crushed and smear on the body. | Ticks, fleas, lice |
| Asclepiadaceae | *Ceropegia convolvuloides* | Merkah | Herb | Fresh whole plant is crushed and allows swallowing. | Antrax /Tafya |
|  | *Ceropegia vignaldiana* | Mshko | Herb | Fresh whole plant is crushed and allows swallowing. | Abdominal pain |
| Asphodelaceae | *Kniphofia isoettfolia* | Ashenda | Herb | Fresh leaf is crushed, mixed with ergo and allow to drink. | Fasciolosis/effel |
| Boraginaceae | *Cynoglossum lanceolatum* | Teng-Begie | Shrub | Fresh leaf is crushed, mixed with honey and smear on affected part of the body. | Wound |
| Colchicaceae | *Gloriosa simplex* | Tslal-enymariam | Herb | Fresh leaf is crushed, mixed with water and smear on the skin of livestock. | Ticks, fleas, lice |
| Euphorbiaceae | *Euphorbia abyssinica* | Kulkale | Shrub | Fresh latex is smear on the affected body. | Body swelling/ zgage |
|  | *Ricinus communis* | Guli | Herb | Fresh leaf is crushed and smear on the affected body. | Wound |
| Fabaceae | *Vigna unguiculata* | Adagura | Herb | Dry seed is powdered, dissolve in milk and allow drinking. | | Fasciolosis |  | | --- | --- |   /effel |
|  | *Crotalaria incana* | Hawwi-leyti | Shrub | Fresh leaf is crushed and allow eating | Diarrhea & shivering /halfyen |
|  | *Acacia abyssinica* | Chiea | Tree | Fresh fruit is crushed, mixed with butter and smear on affected body. | Wound |
| Lamiaceae | *Ocimum basilicum* | Seseg | Herb | Fresh leaf is crushed and allows eating. | Abdominal pain |
|  | *Becium obovatum* | Tehag | Herb | Fresh grass is crushed, mixed with malt and allow swallowing. | Diarrhea |
| Melianthaceae | *Bersama abyssinica* | Asha-om | Shrub | Fresh leaf is crushed, mixed in water and smear on cattle bodies. | Ticks, fleas, lice |
| Moraceae | *Ficus glumosa* | Chekente | Tree | Dry seed is powdered, dissolve in water and allow dinking. | Newcastle disease |
| Poaceae | *Sorghum bicolor* | Mashla | Herb | Dry seed is powdered, mixed with aloe and allow swallowing. | Antrax /Tafya |
| Solanaceae | *Nicotiana tabacum* | Tnbako | Herb | Fresh leaf is crushed, mixed with water, filter and sniff through nose | Leech |
|  | *Discopodium penninervium* | Gaeta | Shrub | Fresh leaf is crushed and allows eating. | Diarrhea & shivering /halafyen/ |
|  | *Capsicum frutescens* | Mitmita | Herb | Fresh fruit is crushed and allow swallowing. | Bloating |
|  | *Capsicum annuum* | Berber | Herb | Dry seed is powdered, dissolve in water and allow drinking. | Bloating/ kebdi mnfah |
