# Supplementary material for: Ethnobotanical study of medicinal plants in Ganta Afeshum District, Eastern Zone of Tigray, Northern Ethiopia
Source: J Ethnobiol Ethnomed. 2018 Nov 3;14:64. doi: 10.1186/s13002-018-0266-z (PMC6215673; doi:10.1186/s13002-018-0266-z)
Supplement: Supplementary file 5 — Table S5. List of medicinal plants used for treating human and livestock ailments. (DOC 164 kb) [file 13002_2018_266_MOESM5_ESM.doc]

Table S5. List of medicinal plants used for treating human and livestock ailments: (Hu-human, Liv-livestock)

| **Family name** | **Scientific name** | **Local name** | **Habit** | **Uses** | **Method of preparation and application; condition of preparation and part used** | **Ailments** |
| --- | --- | --- | --- | --- | --- | --- |
| Acanthaceae | *Justicia schimperiana* | Shimeza | Shrub | Hu | Fresh leaf is crushed, mixed with water, filter and drink. | Jaundice/ Efshwa |
|  |  |  |  | Liv | Fresh leaf is boiled in water and allowed to drink. | Cough |
| Alliaceae | *Allium sativum* | Tsaeda-shgurti | Herb | Hu | Fresh bulb is crushed, mixed with honey and eat at morning for seven days. | Asthma |
|  |  |  |  | Liv | Fresh bulb is crushed, mixed with water, filter and sniff to the nose. | Horn worm & leech |
| Aloaceae | *Aloe camperi* | Sandaere | Herb | Hu | Fresh latex is directly dropped in eye. | Eye disease |
|  |  |  |  | Liv | Fresh latex is smeared to affected skin of oxen especially on the neck. | Wound |
| Aloeaceae | *Aloe megalacantha* | Ere | Herb | Hu | Fresh fruit is crushed, mixed with water and smeared in the anus. | Hemorrhoid |
|  |  |  |  | Live | Fresh latex is allowed to drink | Malaria & Newcastle disease |
|  |  |  |  |  | Fresh latex is smear on the body of livestock. | Ticks |
|  |  |  |  |  | Dry bark is burnt in fire and fumigate by its smoke. | Evil spirit/zarti |
| Amaranthaceae | *Amaranthus caudatus* | Mendef-Adgi/ Hamli-adgi) | Shrub | Hu | Chewing fresh root can treat for teeth ach | Teeth ach |
|  |  |  |  | Liv | Fresh leaf is crushed, adds to water, filter and dropped in ear. | Evil spirit/zarti |
| Anacardiaceae | *Schinus molle* | Tkur-berbere | Tree | Hu | Fresh leaf is crushed, mixed with water, filtered and drink at the time of pain. | Jaundice, Tape worm, |
|  |  |  |  | Liv | Fresh leaf is crushed, mixed with water, filter and sniff through nose | Leech |
| Apiaceae | *Foeniculum vulgare* | Shlan | Herb | Hu | Fresh leaf is crushed, mixed with water, filter and drink. | Urine retention |
|  |  |  |  | Liv | Fresh stem is crushed, adds to water and allowed to drink. | Urine retention |
|  |  |  |  |  | Fresh leaf is crushed and allowed to eat. | Abdominal pain |
| Amaranthaceae | *Achyranthes aspera* | Mchelo | Herb | Hu | Fresh leaf and stem is crushed and smear on head. | Tonsillitis |
|  |  |  |  | Liv | Fresh leaf and stem is crushed, add water, filter by using cotton and dropped in to eye. | Eye disease |
| Asparagaceae | *Asparagus africanus* | Kesta-Ansti | Shrub | Hu | Dry and fresh root is burnt in fire and fumigate by its smoke. | Evil eye |
|  |  |  |  | Liv | Fresh root is crushed, and smear on the affected body. | Body swelling |
| [Asteraceae](https://en.wikipedia.org/wiki/Asteraceae) | *Artemisia abyssinica* | ChenaBarya/wedwado | Herb | Hu | Fresh and dry stem is brunt and fumigate by its smoke. | Evil eye. |
|  |  |  |  | Liv | Fresh leaf is crushed, mixed in water, filter and sniff through nose. | Evil spirit/zarti |
|  | *Vernonia amygdalina* | Grawa | Shrub | Hu | Dry leave is powdered, mixed with honey(brzi) and drink | Impotence in male |
|  |  |  |  | Liv | Fresh leaf is crushed, mixed in water and allowed to drink. | Bloating |
|  | *Laggera tomentosa* | Konshkonsho | Shrub | Hu | Fresh leaf is boiled in water and fumigate by its steam | fibril illness |
|  |  |  |  | Liv | Fresh leaf is crushed, mixed in water and sniff through the nose. | Leech |
| Bignoniaceae | *Stereospermum kunthianum* | Adgizana | Shrub | Hu | Dry bark is crushed and powdered, mix with honey then smeared on affected dermis. | Wound |
|  |  |  |  | Liv | Dry whole plant is crushed, burnt in fire and fumigate by its smoke. | \| Black leg/ \| Wekie \| \| --- \| --- \|   /Wekei/ |
| Boraginaceae | *Cordia africana* | Awhi | Tree | Hu | Fresh leaf is crushed, mixed with tea/coffee and drink. | Fibril illness |
|  |  |  |  | Liv | Fresh leaf is crushed, mixed in water, filter and sniff through nose. | Leech |
| Brassicaceae | *Brassica carinata* | Adri/senafich | Herb | Hu | Dry seed is powdered; mix with water and then drink. | Constipation & blood pressure |
|  |  |  |  | Liv | Dry seed is powdered; mix with water and then allow drinking. | Newcastle disease |
|  | *Lepidium sativum* | Shnfae | Herb | Hu | Dry seed is powdered, mixed with ergo and the drink. | Bloody diarrhea |
|  |  |  |  | Liv | Dry seed is powdered, adds to water and allowed to drink. | Newcastle disease |
| Celastraceae | *Maytenus senegalensis* | Argudi | Shrub | Hu | Fresh leaf is crushed, mixed with butter and smeared in and around anus. | Hemorrhoids |
|  |  |  |  | Liv | Fresh leaf is crushed, mixed in water and allow drinking | Newcastle disease |
| Compositae | *Psiadia punctulata* | Alakit | Shrub | Hu | Fresh leaf is crushed, mixed with *Allium sativum* and smeared on infected dermis. | Herpes/ Almaz balechra/ |
|  |  |  |  | Liv | Fresh leaf and stem used to tie in bone fracture. | Bone fracture. |
| Cucurbitaceae | *Zehneria scabra* | Hafaflo | Shrub | Hu | Fresh leaf is boiled in water and fumigate by its smoke. | fibril illness & cough |
|  |  |  |  | Liv | Fresh leaf is crushed, mixed in water and allowed to drink. | Bloating |
| Cuppressaceae | *Juniperus procera* | Tshdi-habesha | Tree | Hu | Dry seed is powdered, mixed with water and honey and drink. | Fear & dislike of sex in females |
|  |  |  |  | Liv | Fresh leaf is crushed, mixed in water and allowed to drink | Diarrhea& shivering |
| Dracaenaceae | *Sansevieria ehrenbergii* | Eka | Shrub | Hu | Fresh bark is heated in fire and press on the affected body. | Nasal disease |
|  |  |  |  | Liv | Fresh bark is crushed and smear on affected body. | Wound |
| Ebenaceae | *Euclea racemosa* | Kliaw | Shrub | Hu | Fresh and dry root is crushed, mixed with butter and smear on face. | black spot on face/ Madyat |
|  |  |  |  | Liv | Fresh leaf is crushed, mixed in water and sniff through the nose. | Leech |
| Fabaceae | *Acacia mellifera* | kerets | Tree | Hu | Dry root is powdered, mixed with honey and eat. | Lepros*y* |
|  |  |  |  | Liv | Fresh root is crushed, mixed with water and sniff through nose. | Leech |
|  | *Albizia gummifera* | Sasa | Shrub | Hu | Fresh leaf is boiled in water and fumigate by its steam. | Fibril illness |
|  |  |  |  | Liv | Fresh leaf is crushed, adds to water and sniff through nose. | Horn worm/ haseka resi |
|  | *Calpurnia aurea* | Htsawts | Shrub | Hu | Fresh leaf is crushed with water, filter by using cotton and dropped in sick eye. | Eye disease |
|  |  |  |  | Liv | Fresh leaf is crushed and smear on the body. | Fleas & lice |
|  | *Cicer cuneatum* | shmbra-gwasot | Herb | Hu | Chewing fresh root is a treatment for abdominal pain | Abdominal pain |
|  |  |  |  | Liv | Fresh root is crushed, mixed with water, filter and sniff through the nose. | Evil eye |
| Lamiaceae | *Otostegia integrifolia* | Chendog | Shrub | Hu | Fresh leaf is crushed, mixed with water and drink. | Amoeba |
|  |  |  |  | Liv | Fresh leaf is crushed and smear on the skin of cattle. | Ticks |
|  | *Meriandra dianthera* | Meseguh | Shrub | Hu | Fresh leaf is crushed, filter and drink. | Abdominal pain |
|  |  |  |  | Liv | Fresh leaves are crushed, add water and allowed to drink. | Abdominal pain |
| Linaceae | *Linum usitatissimum* | Entatie | Herb | Hu | Dry seed is powdered, mixed with water, boil and drink. | Constipation |
|  |  |  |  | Liv | Dry seed is soak in water for three days and allowed to eat at the delivery time. | Prolonged delivery |
| Lobeliaceae | *Lobelia giberroa* | Grhan | Shrub | Hu | Dry leaf is powdered and sniffed through nose. | Evil eye |
|  |  |  |  | Liv | Fresh leaf is crushed, mixed in water and allowed to drink. | Diarrhea & shivering |
| Malvaceae | *Malva verticillata* | Lhtit | Herb | Hu | Fresh leaf is crushed, filtered and dropped in to ear. | Ear infection |
|  |  |  |  | Liv | Dry and fresh of the whole plant is burnt in fire and fumigate by its smoke. | Evil spirit/zarti |
|  | *Sida schimperiana* | Tfrerya | Shrub | Hu | Chewing fresh root at time of pain is a treatment for | Abdominal pain |
|  |  |  |  | Liv | Unprocessed fresh and dry root tie on the affected part of body. | Bone dislocated |
| Meliaceae | *Melia azedarach* | Niem | Tree | Hu | Fresh leaf is crushed, mixed with water and drink. | Malaria |
|  |  |  |  | Liv | Fresh leaf is crushed, adds to water and allowed to drink. | Bloating |
| Moraceae | *Ficus palmata* | Beles/demay | Tree | Hu | Fresh and dry root is heated in fire and pressed on affected skin. | Body swelling |
|  |  |  |  | Liv | Fresh latex is allowed to drink. | Newcastle disease |
| Myricaceae | *Myrica salicifolia* | Nebi | Tree | Hu | Dry bark is powdered and sniff through nose. | Tumor and head ach. |
|  |  |  |  | Liv | Fresh bark is boiled in water and allowed to drink. | Diarrhea |
| Myrtaceae | *Syzygium guineense* | Liham | Tree | Hu | Fresh bark is boiled in water and drink at morning before eating food. | Diarrhea |
|  |  |  |  | Liv | Fresh bark is boiled and cooked and then allowed to eat. | Diarrhea |
| Oleaceae | *Jasminum abyssinicum* | Habitselim | Shrub | Hu | Fresh leaf is grind, filtered by using cotton and dropped in eye. | Eye disease |
|  |  |  |  | Liv | Fresh leaf is crushed mixed in water, filter and dropped into eye | Eye disease |
| Phytolaccaceae | *Phytolacca dodecandra* | Shbti | Shrub | Hu | Fresh root is crushed, mixed with tella and drink. | Rabies |
|  |  |  |  | Liv | Fresh leaf is crushed, mixed in water and allowed to drink. | Leech |
| Plumbaginaceae | *Plumbago zylanica* | Aftuh | Shrub | .Hu | Fresh leaf is crushed, mix with water and wash every morning for seven days. | Evil eye |
|  |  |  |  | Liv | Fresh root is crushed and smear on the affected body. | Body swelling |
| Poaceae | *Hordeum vulgare* | Sgem/bukuli | Herb | Hu | Dry seed is roasted and eat as food. | Gastritis |
|  |  |  |  | Liv | Dry seed is allowed to soak in water for three days and then allowed to eat. | Bloating |
| Po1ygonaceae | *Rumex nervosus* | Hihot | Shrub | Hu | Fresh root is crushed, add to teji and drink | Impotence in male |
|  |  |  |  | Liv | Fresh is crushed mixed in water and allowed to drink. | Abdominal pain |
|  | *Rumex nepalensis* | Shenbwaeta | Shrub | Hu | Fresh leaf is heated in fire and the rub by the heated leaf on infected part of leaf. | Ring worm |
|  |  |  |  | Liv | Fresh root is crushed and allowed to eat. | Diarrhea &shivering |
| Ranunculaceae | *Dichrostachys cinerea* | Gonek | Shrub | Hu | Fresh bark is tied on damaged part of the body with cotton that never washed before. | Dislocated bone |
|  |  |  |  | Liv | Fresh bark is tied on fractured bone to repair it. | Bone fracture |
| Rhamnaceae | *Rhamnus prinoides* | Gesho | Shrub | Hu | Chewing fresh, young part of the medicinal plant is treatment for tonsillitis | Tonsillitis |
|  |  |  |  | Live | Fresh leaf is crushed, mixed in water, filter, and sniff through nose. | Leech |
|  |  |  |  |  | Fresh leaf is crushed, mixed with butter and allowed to swallow. | Diarrhea & shivering |
| Rutaceae | *Ruta chalepensis* | Chena-Adam | Herb | Hu | Fresh leaf is directly added in tea, coffee, milk and drink. | Cough |
|  |  |  |  | Liv | Fresh leaf is crushed, mixed in water, filter and sniff through nose. | Horn worm/ haseka resi |
|  | *Citrus limon* | Lemin | Shrub | Hu | Fresh fruit is squeezed and rubbed on affected skin. | Skin problem |
|  |  |  |  | Liv | Fresh and dry bark is crushed, mixed with butter and then allowed to eat. | Rabies |
| Sapotaceae | *Sideroxylon oxyacanthum* | Seroro | Tree | Hu | Dry fruit is powdered, heat on oven, mixed with butter and finally smear. | Skin rash |
|  |  |  |  | Liv | Fresh leaf is crushed, mixed with water and allowed to drink. | Horn worm |
| Scrophulariaceae | *Verbascum sinaiticum* | Trnaka | Tree | Hu | Fresh root is crushed, mixed with water, filtered and drink. | Retained placenta |
|  |  |  |  | Liv | Fresh root is crushed and smear on affected body. | Body swelling |
| Solanaceae | *Withania somnifera* | Agol | Shrub | Hu | Fresh leaf is boiled in water and fumigate by its steam | fibril illness |
|  |  |  |  | Liv | Fresh leaf is crushed, mixed in water and allowed to drink. | Bloating |
|  | *Nicandra physalodes* | Hamli –kbo | Herb | Hu | Dry leaf is powdered; mixed with water, smear on the damaged skin. | Fire Burn Wound |
|  |  |  |  | Liv | Fresh leaf is crushed, mixed with butter and allowed to swallow. | Diarrhea & shivering |
|  | *Solanum incanum* | Engule | Shrub | Hu | Dry root is crushed, mixed with honey and eat. | Leprosy |
|  |  |  |  | Live | Fresh leaf is crushed, mixed in water allowed to drink. | Diarrhea & shivering |
|  |  |  |  |  | Fresh fruit is crushed, squeezed-add to water and allowed to drink. | Leech |
|  |  |  |  |  | Fresh fruit is allowed to eat. | Abdominal pain |
|  | *Lycopersicon esculentum* | Kumedre | Herb | Hu | Fresh leaf is crushed and smeared on head. | Tonsillitis |
|  |  |  |  | Liv | Fresh leaf is crushed, mixed in water and sniff through the nose. | Leech |
|  | *Datura stramonium* | Mezerbae | Herb | Hu | Dry fruit is roasted on oven with butter and fumigate by its smoke. | Teeth ach |
|  |  |  |  | Liv | Fresh leaf is crushed, mixed with butter and allowed to eat. | Diarrhea & shivering |
| Sterculiaceae | *Dombeya torrida* | Tsnkuya | Shrub | Hu | Fresh bark is crushed and smear on affected body. | Fire Burn Wound |
|  |  |  |  | Liv | Fresh leaf is boiled in water and allowed to drink. | Prolonged delivery |
| Verbenaceae | *Clerodendron myricoides* | Surbetry | Shrub | Hu | Fresh leaf and stem is burnt in fire and fumigate by its smoke. | Feberileillne |
|  |  |  |  | Liv | Fresh stem is crushed and smear on affected body. | Body swelling |
